# Energy Transfer Mechanism and Quantitative Modeling of Rate from an Antenna to a Lanthanide Ion

**DOI:** 10.1021/acs.jpca.2c03965

**Published:** 2022-10-06

**Authors:** Peter A. Tanner, Waygen Thor, Yonghong Zhang, Ka-Leung Wong

**Affiliations:** † Department of Chemistry, Hong Kong Baptist University, Waterloo Road, Kowloon Tong, Hong Kong S.A.R., P. R. China; ‡ State Key Laboratory of Chemistry and Utilization of Carbon Based Energy Resources, Key Laboratory of Oil and Gas Fine Chemicals, Ministry of Education & Xinjiang Uygur Autonomous Region, Urumqi Key Laboratory of Green Catalysis and Synthesis Technology, College of Chemistry, Xinjiang University, Urumqi 830017 Xinjiang, P. R. China

## Abstract

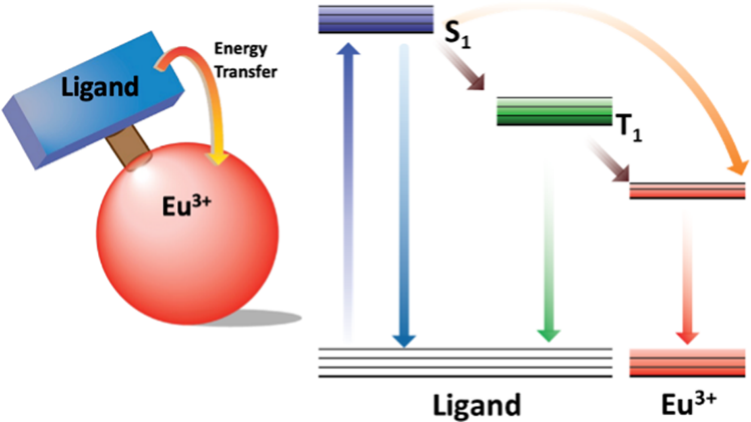

The excitation energy transfer (ET) pathway and mechanism from an organic antenna to a lanthanide ion has been the subject of discussion for many decades. In the case of europium (Eu^3+^), it has been suggested that the transfer originates from the ligand singlet state or a triplet state. Taking the lanthanide complex **Eu(TTA)**_**3**_**(H**_**2**_**O)**_**2**_ as an example, we have investigated the spectra and luminescence kinetics, mainly at room temperature and 77 K, to acquire the necessary experimental data. We put forward an experimental and theoretical approach to measure the energy transfer rates from the antenna to different Eu^3+^ levels using the Dexter formulation. We find that transfer from the ligand singlet state to Eu^3+^ may account for the ET pathway, by combined electric dipole–electric dipole (ED–ED) and ED-electric quadrupole (EQ) mechanisms. The contributions from the triplet state by these mechanisms are very small. An independent systems rate equation approach can effectively model the experimental kinetics results. The model utilizes the cooperative processes that take place on the metal ion and ligand and considers S_0_, S_1_, and T_1_ ligand states in addition to ^7^F_0,1_, ^5^D_0_, ^5^D_1_, and ^5^DJ (=^5^L_6_, ^5^D_3_, ^5^D_2_ combined) Eu^3+^ states. The triplet exchange ET rate is estimated to be of the order 10^7^ s^–1^. The observation of a nanosecond risetime for the Eu^3+ 5^D_1_ level does not enable the assignment of the ET route or the mechanism. Furthermore, the ^5^D_1_ risetime may be contributed by several processes. Observation of its temperature dependence and also that of the ground-state population can supply useful information concerning the mechanism because the change in metal-ion internal conversion rate has a greater effect than changes in singlet or triplet nonradiative rates. A critical comparison is included for the model of Malta employed in the online software LUMPAC and JOYSpectra. The theoretical treatment of the exchange mechanism and its contribution are now being considered.

## Introduction

**Figure 1:**
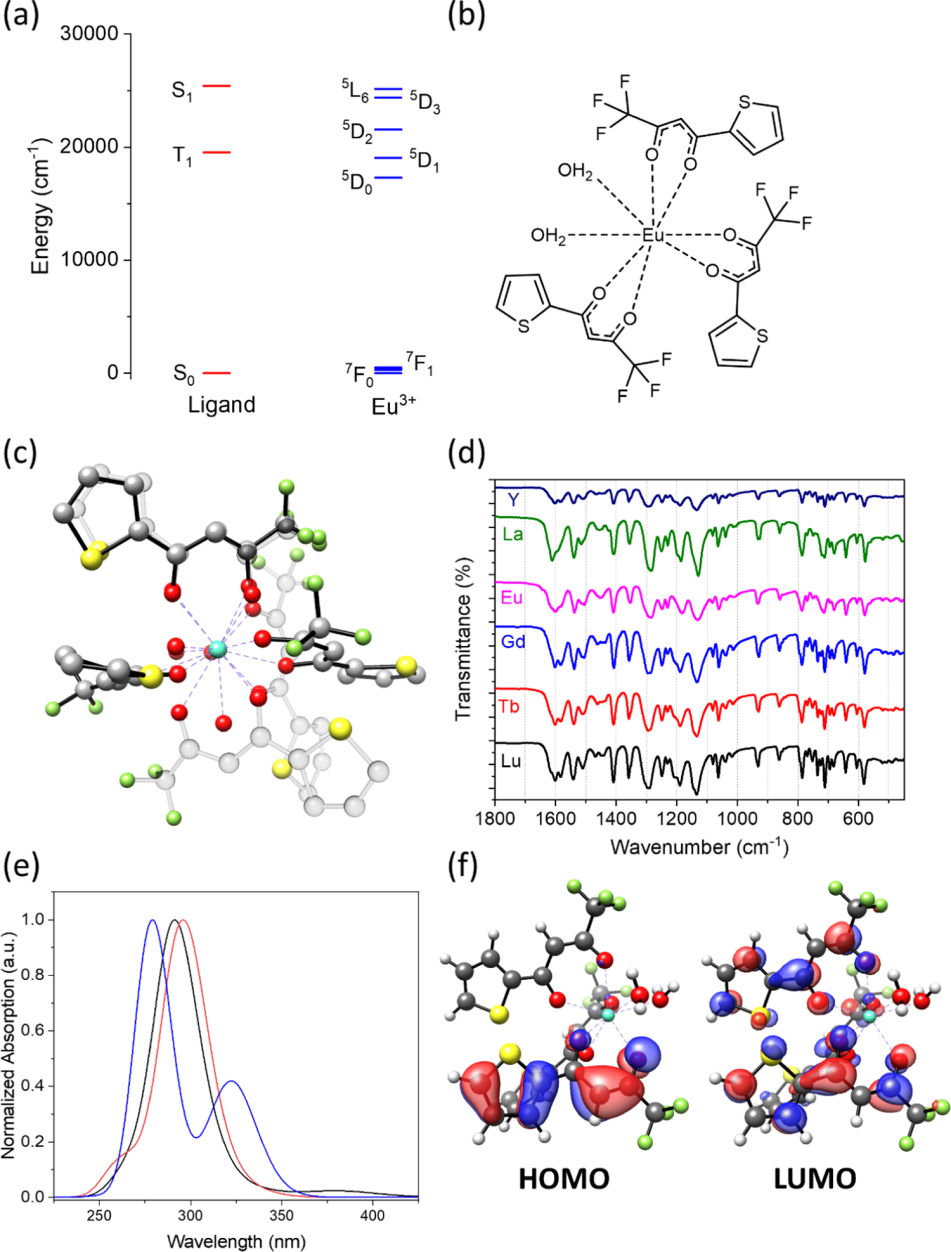
(a) Selected energy levels of the ligand-Eu^3+^ system. (b) Schematic structure of the complex. (c) X-ray structure of triclinic Isomer I of **Eu(TTA)**_**3**_**(OH**_**2**_**)**_**2**_ showing the disorder of the thienyl group in one of the coordinated TTA ligands^14^ (black bonds) superimposed upon Isomer II, the monoclinic structure^15^ (gray bonds). (d) Infrared spectra of **Ln(TTA)**_**3**_**(OH**_**2**_**)**_**2**_ complexes. (e) Calculated ultraviolet absorption spectra using time-dependent density functional theory (TD-DFT) for **Eu(TTA)**_**3**_**(OH**_**2**_**)**_**2**_ in the gas phase (black) and in toluene solution (red). The MWB52-PBE0/D3BJ/def2-TZVP level of theory was employed, and the CPCM model was employed for the solution spectrum. The CASSCF-NEVPT2 calculated spectrum using a (6,6) active space is shown in blue. (f) DFT frontier orbitals of **Eu(TTA)**_**3**_**(OH**_**2**_**)**_**2**_ in toluene solution.

Lanthanide ions are versatile lighting and display elements, but their performance relies upon an efficient antenna because their absorption is very weak. Hence, there have been many thousands of papers describing the quantum efficiency and brightness of their complexes with organic ligands, particularly for europium, Eu^3+^. From experiment, some studies have proposed that the singlet state S_1_ (or a charge transfer (CT) state) is responsible for the transfer of energy from the coordinated ligand to the Eu^3+^ energy levels,^1−5^ whereas the majority of reports consider that energy transfer (ET) occurs from the ligand triplet state, T_1_^6−8^ (Figure 1a). The understanding of the major ET pathway and mechanism is particularly important for the development and optimization of photovoltaic and optoelectronic devices, such as in lighting and displays, light-emitting diodes (LEDs), biomarkers, sensors, fluoroimmunoassays, and noncontact thermometers.^9−13^

Naturally, the Eu^3+^ population mechanism can depend upon many factors including the type of ligand system. However, confirmatory evidence from experiment should show that the Eu^3+^ acceptor luminescence risetime is equal to the *donor* decay time. Moreover, even the confirmation of the energy transfer route may not be sufficient to elucidate the relevant mechanism.^6^

Hence, attempts to unravel the population channel and its mechanism have turned to theory. However, to date, there has only been a comprehensive quantitative description given by Malta and co-workers^12,16−18^ for the transfer of energy from the antenna to the Eu^3+^ metal ion. The model of Malta, using the tensor operator techniques of Kushida,^19^ has been employed in many publications and has sometimes concluded that singlet ET occurs to Eu^3+^, and in other cases, triplet ET occurs. The earlier publications evidently overestimated the contributions from exchange ET. The model is available for use in free and widely used computation software programs called LUMPAC^20^ and JOYSpectra.^21^

The key question which has been targeted in many publications is: does the antenna ET originate from a singlet or a triplet channel? Naturally, both can contribute to varying extents. We present an alternative strategy for attacking this question which utilizes experimental data in conjunction with theory. The compound **Eu(TTA)**_**3**_**(H**_**2**_**O)**_**2**_ (TTA = thenoyltrifluoroacetonate) (Figure 1b) has been chosen for the demonstration of the results because it has been widely studied and applied,^8,22−28^ and it has been used for illustration in the software JOYSpectra.^21^ The temporal variation of the populations of ligand and metal states has been reproduced by a new rate equation model.

## Methods

### Materials

**EuCl**_**3**_**·6H**_**2**_**O** and **LaCl**_**3**_**·7H**_**2**_**O** were purchased from Sigma-Aldrich (≥99.99% trace-metal basis) and used without further purification. All organic chemicals were purchased from TCI or Energy Chemical with 98% purity and used without further purification.

### Synthesis

**Ln(TTA)**_**3**_**(H**_**2**_**O)**_**2**_ were obtained according to a procedure following the literature method.^24^ Briefly, for Ln = Eu, **HTTA** (0.67 g, 3 mmol) was dissolved in 15 mL of ethanol in a 150 mL flask, with stirring at room temperature. Then, the pH of the solution was adjusted to 6–7 by the addition of **NaOH** solution (1.0 M). After that, 1 mmol of **EuCl**_**3**_**·6H**_**2**_**O** (0.37 g, 1 mmol) solution in 5.0 mL of deionized water was added to the above mixture at 60 °C. Then, deionized water (100 mL) was added to the above mixture with vigorous stirring for 2 h at 60 °C to ensure complete precipitation. After cooling to room temperature, the precipitate was filtered, washed repeatedly with water, and dried overnight under vacuum at room temperature to afford **Eu(TTA)**_**3**_**(H**_**2**_**O)**_**2**_ as a light yellow solid (0.68 g, 80%). **Ln(TTA)**_**3**_**(H**_**2**_**O)**_**2**_ (Ln = Gd, La) were prepared analogously. The compound easily decomposes in a moist atmosphere. The results of thermogravimetric analysis are shown in Figure S1.

### Instruments

Excitation and emission spectra and luminescence decay curves were obtained by a Fluorolog-3 spectrometer from Horiba with a 450 W Xenon lamp, Horiba Nano, and Spectra LEDs as excitation sources. Spectra were recorded at 77 K with the samples in NMR tubes in a home-built liquid nitrogen setup made of glass. Experiments at the sensor reading of 10 K (i.e., the nominal 10 K temperature) were performed using an Optical Cryostat-CS202I-DMX-1SS from Advanced Research Systems Instruments, Inc. Emission spectra with higher resolution were also measured using an iHR 550 spectrometer with a system consisting of a Nd:YAG pump laser, a third-order harmonic generator (THG at 355 nm, 120 mJ), and an optical parameter oscillator (OPO, Spectra-Physics VersaScan, and UVScan) with a pulse duration of 8 ns and repetition frequency of 10 Hz. Absorption spectra were recorded at room temperature for solutions in quartz cuvettes using a PerkinElmer LAMBDA 1050+ UV/VIS/NIR double-beam spectrophotometer. A qX3/Horiba4 variable-temperature cell was employed to investigate the temperature dependence of emission between 280 and 323 K. A 10 mm pathlength cuvette was used for all room-temperature measurements. Fourier transform infrared (FT-IR) spectra were recorded using a PerkinElmer FT-IR Spectrum Two equipped with LiTaO_3_ detector.

### Computation of Molecular Structure and State Energies. Rate Equations

Calculations were performed using Orca version 4.2.1^29,30^ and accessories, together with the use of Avogadro^31,32^ and Gabedit.^33^ In these density functional theory (DFT) calculations, the PBE0^34,35^ functional was employed with the def2-TZVP basis set,^36^ with the Stuttgart in-core effective core potential and basis set for Eu^3+^.^37,38^ In all optimizations, the Grimme dispersion correction D3BJ was used.^39,40^ The calculation for **Eu(TTA)**_**3**_**(H**_**2**_**O)**_**2**_ in toluene solvent used the Conductor-like Polarizable Continuum Model (CPCM).^41^ The optimized structures were checked to represent true minima by calculation confirming the absence of imaginary frequencies. The simulated absorption spectra employed a bandwidth of 2000 cm^–1^. The time-dependent DFT calculation employed 30 roots. Molecular orbital figures used the isosurface values of 0.05 *e*/*a*_0_^3^ in all cases.

CASSCF-NEVPT2 calculations^42−44^ were also performed using ORCA 4.2.1. for 10 roots, following a second-order Møller–Plesset theory calculation with the optimized structure from the above DFT. The basis set 6-31+G(d)^45^ was used for all atoms except Eu, with the auxiliary basis: def2-TZVP/C.^46^

Multiwfn v3.7.^47^ was used to determine the molecular orbital coefficients at atomic positions. Distances from Eu to the relevant atoms were measured in Diamond^48^ and Avogadro.^31,32^

The wavefunction for ^7^F_0_ (or ^5^D_0_, for example) comprises several other SLJ multiplets in the intermediate coupling scheme. For example, for Eu^3+^ in **Lu**_**2**_**O**_**3**_, the wavefunction of ^7^F_0_ due to *J*-mixing is^49^
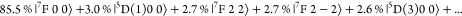
where the two numbers after the term represent *J* and *M*_*J*_ values. Hence, there is about a 5% contribution to the ^7^F_0_ wavefunction from ^7^F_2_ and this has been recognized in the reduced matrix elements given by Kasprzycka et al.^50^ Ideally, this contribution should be included in calculations, but for simplicity, we restrict our calculations to mainly order-of-magnitude results.

Maple 2021^51^ was employed for computations involving the rate equation model.

## Theoretical Background

The oscillator strength *P*_*if*_ of a transition between initial (*i*) and final (*f*) states of a system is measured experimentally from an emission spectrum if the low-temperature lifetime *τ* is approximated as the radiative lifetime *τ*_R_, as^52,53^
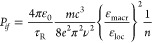
1aor
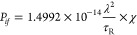
1bwhere the virtual cavity model correction for the macroscopic and local electric field has been inserted in (eq 1b) for an electric dipole (ED)-allowed transition  or χ_MD_ = *n*^3^ for a magnetic dipole (MD)-allowed transition; ε_0_ (F m^–1^) is the vacuum permittivity, *ν* (s^–1^) is the transition frequency, *c* is the speed of light (m s^–1^), *m* is the electron mass (kg), λ is the emission wavelength (in nm), *n* is the refractive index, and *τ*_R_ (s) = 1/*A* is the radiative lifetime (*A* is the Einstein coefficient of spontaneous emission, s^–1^).

Alternatively, the oscillator strength can be approximately measured from the absorption spectrum: from the linear absorption coefficient α (m^–1^) or the absorption cross section σ (m^2^), where *N* (m^–3^) is the number of absorbing centers per unit volume, and *i* and *f* are the initial and final levels in the transition
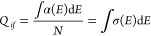
2
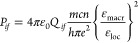
3Here, *h* is Planck’s constant (J s) and *E* = *h*ν. Hence, in terms of ν̅ (cm^–1^)
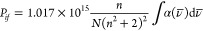
4or in terms of molar absorption coefficient, ε (mol^–1^ cm^–1^ dm^3^)
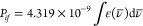
5where
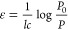
6with *l* being the pathlength (cm), *c* the concentration (mol dm^–3^), and *P*_0_ and *P* the incident and transmitted power through the sample, respectively. The use of eq 5 pertains to the population of the initial state, which for ^7^F_0_ is rather less than 1.

The oscillator strength for an ED-allowed transition can be calculated from the line strength (in C^2^ m^2^), *S*_*if*_ (ED) = ∑_*i,f*_|⟨φ*_f_*|μ_e_|φ_*i*_⟩|^2^ of the transition, where *μ*_e_ is the ED moment operator
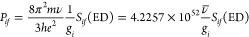
7and *g*_*i*_ is the degeneracy of the initial state (which is usually omitted or often placed in the numerator). Since our cases correspond to nondegenerate (^7^F_0_ and S_1_) initial states, we do not go into detail here. In eq 7, ν̅ is the peak maximum in cm^–1^. For example, from eq 7, if *P*_*if*_ (singlet) = 0.5 at 25 000 cm^–1^, then *S*_*if*_ equals 4.7329 × 10^–58^ C^2^ m^2^ or 4.2537 × 10^–35^ esu^2^ cm^2^. The inclusion of local field effects changes the answer in eq 7 by a factor of ∼3.

The oscillator strengths for the analogous transitions in absorption and emission are related by the state degeneracies
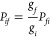
8The energy transfer rate by the ED–ED mechanism between the donor (D) and acceptor (A) separated by distance *R* (see later) can be written
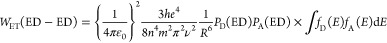
9a
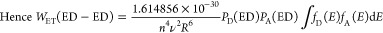
9bwhere the units are *ν* (s^–1^) (the average frequency of the transitions involved), *R* (m), and the overlap integral between the normalized emission and absorption spectra is in J^–1^.

Now we consider interaction comprising an ED transition of the antenna and an electric quadrupole (EQ) transition of the lanthanide ion. The equation given by Dexter for this interaction, neglecting local field effects, is^54^
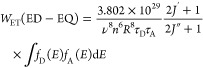
10which shows an *R*^–8^ dependence. In terms of line strengths, this can be written as^52^
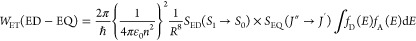
11a
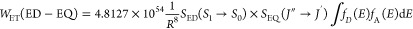
11bwhere the dipole line strength refers to the ligand and the quadrupole line strength to europium, respectively.

The line strength of an EQ transition (in C^2^ m^4^) from lower level *i* to upper level *f* is given by^55^
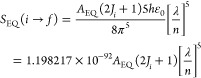
12awhere λ is in nm; or in terms of oscillator strength
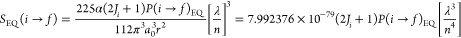
12bwhere α is the fine structure constant, *a*_0_ is the Bohr radius, ⟨*r*^2^⟩ is the expectation value of the radial wavefunction for Eu^3+^, and λ is in nm.

The line strength of a ligand ED transition (in C^2^ m^2^) from an upper level S_1_ to a lower level S_0_ (both with spin *S* = 0) is given by^56,57^
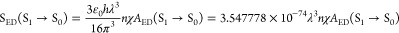
13with λ in nm. For a given transition, the ratio^52,54^
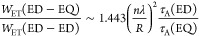
14

Values of *A*_EQ_^′^ = 1/(*n*^5^ × τ_A_(EQ)) for the Eu^3+^ free ion have been tabulated by Dodson and Zia.^55^ Values of *A*_EQ_^′^ for the transitions ^5^D_2_ → ^7^F_0_ at 21 752 cm^–1^ (460 nm) and ^5^G_2_ → ^7^F_0_ at 26 423 cm^–1^ (378 nm) are largest for those under consideration herein, with magnitudes of 3.14 × 10^–3^ and 4.92 × 10^–3^ s^–1^, respectively.

The distance *R* in eqs 9a–11b does not represent the average donor ligand (O)-Eu acceptor distance, but rather it is the electronic barycenter of the donor state, and the coefficients (*c*_*i*_) of the donor singlet or triplet molecular orbital at each atom *i* can be calculated by the Hirshfeld method using Multiwfn 3.7.^47^ The value of *R* is then
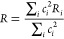
15where *R*_*i*_ is the distance from atom *i* to Eu. We employed the results from our TD-DFT calculation, in addition to a second-order Møller–Plesset (MP2) perturbation calculation, of the orbital compositions of the ligand singlet and triplet states. The calculated values of *R*, in Å, for the singlet state are TD-DFT 3.79, MP2 4.56; and for the triplet state are TD-DFT 3.96, MP2 4.45. The value given in most publications^12^ is 4.5, and we prefer to use this value for reference since it basically agrees with our MP2 calculation results.

Although the free ion selection rules forbid the ED mechanism for the transition (and others) ^5^D_0_ → ^7^F_0_, the transition is usually observed by this mechanism with an oscillator strength of ∼10^–8^ due to the crystal field selection rules incorporating the admixture of other states into the initial and terminal levels. It is therefore considered that the use of eqs 9a and 9b are justified since the Eu^3+^ site symmetry is *C*_1_ in the present case. The contribution of vibronic structure to the intensity of an electronic transition is of paramount importance for high-symmetry systems,^58^ but for Eu^3+^ ions in organometallic systems, most of the spectral intensity arises from pure electronic transitions. Hence, for this reason, in our largely order-of-magnitude calculations, and to avoid unnecessary complication, we do not consider vibronic intensity contributions to spectra. Additionally, we do not consider contributions from magnetic dipole transitions.

## Results and Discussion

### Structure, Frontier Orbitals, and Calculated Spectra of **Eu(TTA)_3_(H_2_O)_2_**

The crystal structure of **Eu(TTA)**_**3**_**(H**_**2**_**O)**_**2**_ was reported by White (monoclinic, *P*2_1_/*c*, *Z* = 4)^15^ and subsequently, for two further isomers (triclinic, *P*1̅, *Z* = 2) by Vallet and co-workers.^14^ The structures are overlaid in Figure 1c, and the reader is referred to the detailed comparison given by Vallet et al. In each case, the site symmetry of the Eu^3+^ ion is *C*_1_ and the coordination number is 8, including two aqua ligands. The FT-IR spectra of the **Ln(TTA)**_**3**_**(H**_**2**_**O)**_**2**_ complexes are shown in Figure 1d. The FT-IR spectrum is a good identification method, and we have compared our spectra with those previously published and with DFT calculation in Table S1.^24,25,59^ We modeled the structure using the ORCA 4.2.1 program,^29,30^ either in the gas phase or in toluene (employing the CPCM),^41^ and the nearest-neighbor bond distances are listed in Table S2, in comparison with those from the crystal structures. The common statistic is the longer distance for Eu–O_w_ bonds.

### Singlet Energy and Oscillator Strength

Our TD-DFT calculations in the gas phase or toluene solution indicate the ultraviolet peak maximum of **Eu(TTA)**_**3**_**(H**_**2**_**O)**_**2**_ at ∼295 nm, Figure 1e, with the frontier orbitals from the gas-phase calculation shown in Figure 1f. The CASSCF-NEVPT2 calculation using a (6,6) active space indicates a longer wavelength, 347 nm. We envisage that after ultraviolet excitation, rapid intersystem crossing occurs from the higher singlet states to the lowest one, which then can undergo intersystem crossing and/or direct ET to Eu^3+^. The transfer occurs from the S_1_–S_0_ zero phonon line (ZPL) energy, and its detailed identification and energy are discussed in the Supporting Information (SI), Section S3. In conclusion, for **Eu(TTA)**_**3**_**(H**_**2**_**O)**_**2**_, we take the zero phonon line energy of the lowest singlet state at 25 410 cm^–1^ (394 nm) and the oscillator strength from 0.005 up to a maximum value of 0.05. We only consider energy transfer to Eu^3+^ levels below this energy, which are above or coincident with the ^5^D_0_ luminescent state. Table S3 lists the Eu^3+^ SLJ multiplet energy levels of several Eu^3+^ systems and shows that in the present ET process, Eu^3+^ could be excited to the ^5^D_*J*_ (*J* = 0–3) and ^5^L_6_ levels, which lie below S_1_ in energy.

### Triplet Energy and Oscillator Strength

**Figure 2:**
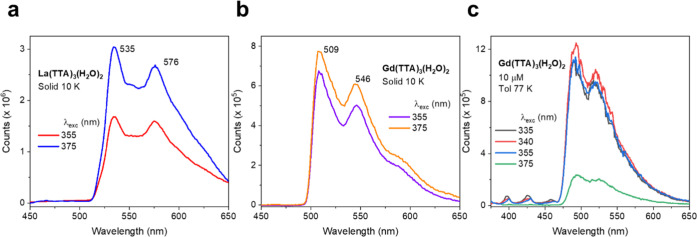
Phosphorescence spectra of solid (a) **La(TTA)**_**3**_**(H**_**2**_**O)**_**2**_ and (b) **Gd(TTA)**_**3**_**(H**_**2**_**O)**_**2**_ at the nominal temperature of 10 K. (c) Fluorescence and phosphorescence spectrum of **Gd(TTA)**_**3**_**(H**_**2**_**O)**_**2**_ in toluene at 10 μM concentration at 77 K. Excitation wavelengths (in nm) were used as indicated. The phosphorescence spectra do not exhibit change in wavelength when the excitation wavelength is altered.

Malta et al.^22^ noted that the phosphorescence emission band in **Gd(TTA)**_**3**_**(H**_**2**_**O)**_**2**_ is not observed in **Eu(TTA)**_**3**_**(H**_**2**_**O)**_**2**_ and concluded that the ET from the lowest triplet state of the TTA ligand to Eu^3+^ is efficient. Other scenarios could be that the triplet level is bypassed and/or energy transfer also occurs from the singlet state. We have measured the low-temperature solid state (Figure 2a,b) and frozen-solution (Figure 2c) phosphorescence spectra of **Ln(TTA)**_**3**_**(H**_**2**_**O)**_**2**_ (Ln = La, Gd). The spectrum of **La(TTA)**_**3**_**(H**_**2**_**O)**_**2**_ solid has a strong 0–0 line at 534.7 nm (18 701 cm^–1^) with the dominant progression frequency of 1349 cm^–1^, corresponding to the unresolved band comprising the antisymmetric C–F stretch and the thiophene ring stretch. The corresponding Gd^3+^ complex, Figure 2b, has a spectrum similar to that previously reported,^23^ but we assign the zero phonon line to the prominent peak at 508.5 nm (19 666 cm^–1^), with the progression frequency of 1350 ± 4 cm^–1^. The difference in zero phonon line energies for Ln = Gd, La is 965 cm^–1^, and from this, we estimate the triplet state energy of **Eu(TTA)**_**3**_**(H**_**2**_**O)**_**2**_ at ∼19 530 cm^–1^ (512 nm). The spectrum of **Gd(TTA)**_**3**_**(H**_**2**_**O)**_**2**_ in toluene at 77 K, Figure 2c, shows a blueshift of the phosphorescence from that in Figure 2b, in addition to the observation of singlet emission.

We have found that due to the magnetic/exchange properties of Gd^3+^, the complexes generally have short phosphorescence lifetimes. Hence, to estimate the (unquenched) triplet state lifetime for **Eu(TTA)**_**3**_**(H**_**2**_**O)**_**2**_, we prefer to use the lifetime for the analogous La complex, which we measured as 0.2 s at 77 K in the solid state. This value is consistent with the reported phosphorescence lifetimes of other lanthanum complexes.^60^ However, the magnitude represents the lowest value for the radiative lifetime, and using eq 1, it gives the highest value for the T_1_–S_0_ transition oscillator strength, *P*_*if*_ ∼ 6.53 × 10^–9^.

### Europium Emission Spectrum and Its Temperature Dependence

**Figure 3:**
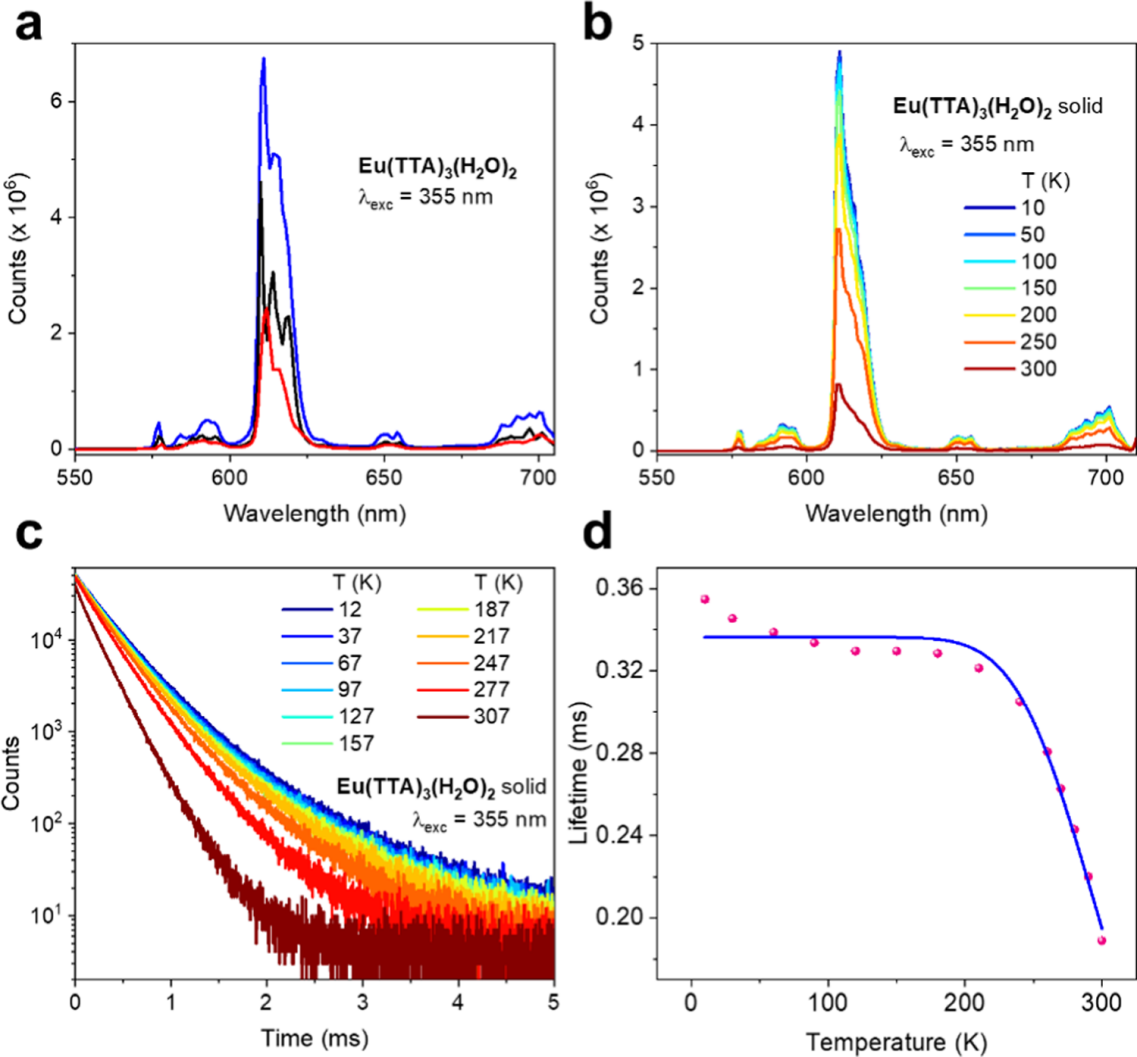
(a) 355 nm Excited room-temperature emission spectra of **Eu(TTA)**_**3**_**(H**_**2**_**O)**_**2**_: blue and black, solid, at different resolution in two instruments; red in toluene solution. The relative intensities are arbitrary for clarity of observation. (b) Temperature dependence of solid-state **Eu(TTA)**_**3**_**(H**_**2**_**O)**_**2**_ emission from 10 to 300 K. (c) Measured ^5^D_0_ luminescence decay curves of solid **Eu(TTA)**_**3**_**(H**_**2**_**O)**_**2**_ at different temperatures and (d) data plot of monoexponential lifetime versus temperature. The blue line follows *y* = 1/(*A* + B* exp(−*C*/0.695 × *x*)), with *A* = 2972 ± 26, *B* = (8.1 ± 8.6) × 10^6^, and *C* = 1716 ± 211. The fit shows that other factors may be involved at lower temperatures (*R*_adj_^2^ = 0.9758).

The room-temperature^22^ and 77 K^23^ luminescence spectra of **Eu(TTA)**_**3**_**(H**_**2**_**O)**_**2**_ have previously been published and assigned. We note, in Figure 3a, that the room temperature spectra differ for the solid state and toluene solution, indicating a different coordination environment for Eu^3+^, presumably due to the loss of water in toluene solution.

Many publications employing the model of Malta have included contributions to ET rates from ^7^F_1_ because at room temperature, the occupation of this *J*-multiplet is appreciable. From emission spectra, the ^7^F_1_ crystal field levels of **Eu(TTA)**_**3**_**(H**_**2**_**O)**_**2**_ are located at 288, 358, and 493 cm^–1^^23^ so that the room temperature population ratio ^7^F_1_/^7^F_0_ is estimated as 0.48 from the barycenter of 380 cm^–1^.

The participation of the ^7^F_1_*J*-multiplet in facilitating ET from an antenna to Eu^3+^ has been invoked in the literature^1,21,28^ because the SLJ selection rules forbid transfer involving ^7^F_0_. The argument is that at room temperature, where the population of ^7^F_1_ has been taken as 0.33, for example, in ref (1), it can act as an effective receiving state. Theoretically, the temperature dependence of Eu^3+ 5^D_0_ population is weak because multiphonon relaxation to the next-lowest level, ^7^F_6_, involves the energy gap of ∼12 020 cm^–1^, which is spanned by many phonons. Hence, if ^7^F_1_ is dominant in participating in the ET process, one would expect an increase in Eu^3+^ emission intensity with temperature. Figure 3b shows that, unless other factors are involved, this is not the case, and that the emission intensity shows a progressive decrease with increasing temperature. The decay of ^5^D_0_ at different temperatures is shown in Figure 3c, and the curves were fitted by monoexponential functions to give lifetimes in the region of 0.3 ms, which are plotted against temperature in Figure 3d and fitted by a single barrier model. The fit gives an activation energy of 1716 ± 211 cm^–1^, which is consistent with the 1749 cm^–1^ gap between ^5^D_0_ and ^5^D_1_ in **Eu(TTA)**_**3**_**(H**_**2**_**O)**_**2**_.

### Oscillator Strengths of Eu^3+^ Transitions

The europium ion transitions are very weak in intensity. Some transitions are observed in solid **Eu(TTA)**_**3**_**(H**_**2**_**O)**_**2**_, as marked in Figure S2, but they are not evident in toluene solution in the same figure. We have measured the oscillator strengths by absorption spectroscopy using eq 5 for data from **Eu(TTA)**_**3**_**(H**_**2**_**O)**_**2**_ in toluene solution and also aqueous **EuCl**_**3**_. The results are listed in Table S4 together with values from other studies.

### Magnitudes of Spectral Overlap Integrals

The integral in eqs 9a, 9b, and 10 is termed the spectral overlap integral. In units of energy^–1^, it measures the overlap of the emission band of the donor moiety with the absorption band of the acceptor. Each band is normalized to unit area. In the present case, the emission from the singlet or triplet state is very broad compared with the sharp intra-4f^6^ absorption transitions. The SI, Section S5, gives an example of the calculation of this integral by representing the absorption and emission bands by Gaussians (Figure S4). The value was directly calculated using Maple 2021 software.^51^ Alternatively, the value was calculated by summation of the intervals in the data columns,^61^ and the answer was in good agreement (Section S5). We have therefore employed this summation method in the present study to calculate spectral overlap integrals (Table 1). Note that the spectral overlap integral does not represent the area bounded under the donor and acceptor curves (Figure S5), which gives a much smaller answer.

**Table 1:** Calculated Spectral Overlap Integrals for Various Scenarios

system^a^	transitions	SO (eV^–1^)	Figures
GdTTA phos + EuTTA abs	T_1_ → S_0_, ^7^F_0_ → ^5^D_1_	2.324	
GdTTA phos + EuCl_3_ abs	T_1_ → S_0_, ^7^F_0_ → ^5^D_1_	2.254	4a
GdTTA phos + EuTTA em	T_1_ → S_0_, ^5^D_0_ → ^7^F_0_	1.141	4b
GdTTA phos + EuTTA em	T_1_ → S_0_, ^5^D_0_ → ^7^F_0_	1.279	S6a
GdTTA em + EuTTA abs	S_1_ → S_0_, ^7^F_1_ → ^5^D_1_	0.494	4c
GdTTA phos + EuTTA abs	T_1_ → S_0_, ^7^F_1_ → ^5^D_1_	2.317	4d
HTTA em + EuTTA em	S_1_ → S_0_, ^5^D_0_ → ^7^F_0_	0.232	S6b
GdTTA em + EuTTA em	S_1_ → S_0_, ^5^D_0_ → ^7^F_0_	0.197	S6c
GdTTA em + EuTTA abs	S_1_ → S_0_, ^7^F_0_ → ^5^D_1_	0.550	S6d
HTTA em + EuCl_3_ abs	S_1_ → S_0_, ^7^F_0_ → ^5^D_1_	0.619	S6e
HTTA em + EuCl_3_ abs	S_1_ → S_0_, ^7^F_0_ → ^5^D_2_	1.754	S6f
GdTTA em + EuTTA exc	S_1_ → S_0_, ^7^F_0_ → ^5^D_2_	1.326	S6g
GdTTA em + EuTTA abs	S_1_ → S_0_, ^7^F_0_ → ^5^D_2_	1.354	S6h
GdTTA em + EuCl_3_ abs	S_1_ → S_0_, ^7^F_0_ → ^5^D_3_	1.292	S6i
GdTTA em + EuCl_3_ abs	S_1_ → S_0_, ^7^F_0_ → ^5^L_6_	0.172	S6j

a LnTTA = **Ln(TTA)**_**3**_**(H**_**2**_**O)**_**2**_ (Ln = Gd, Eu); phos = phosphorescence spectrum; abs = absorption spectrum; em = emission spectrum; exc excitation spectrum.

**Figure 4:**
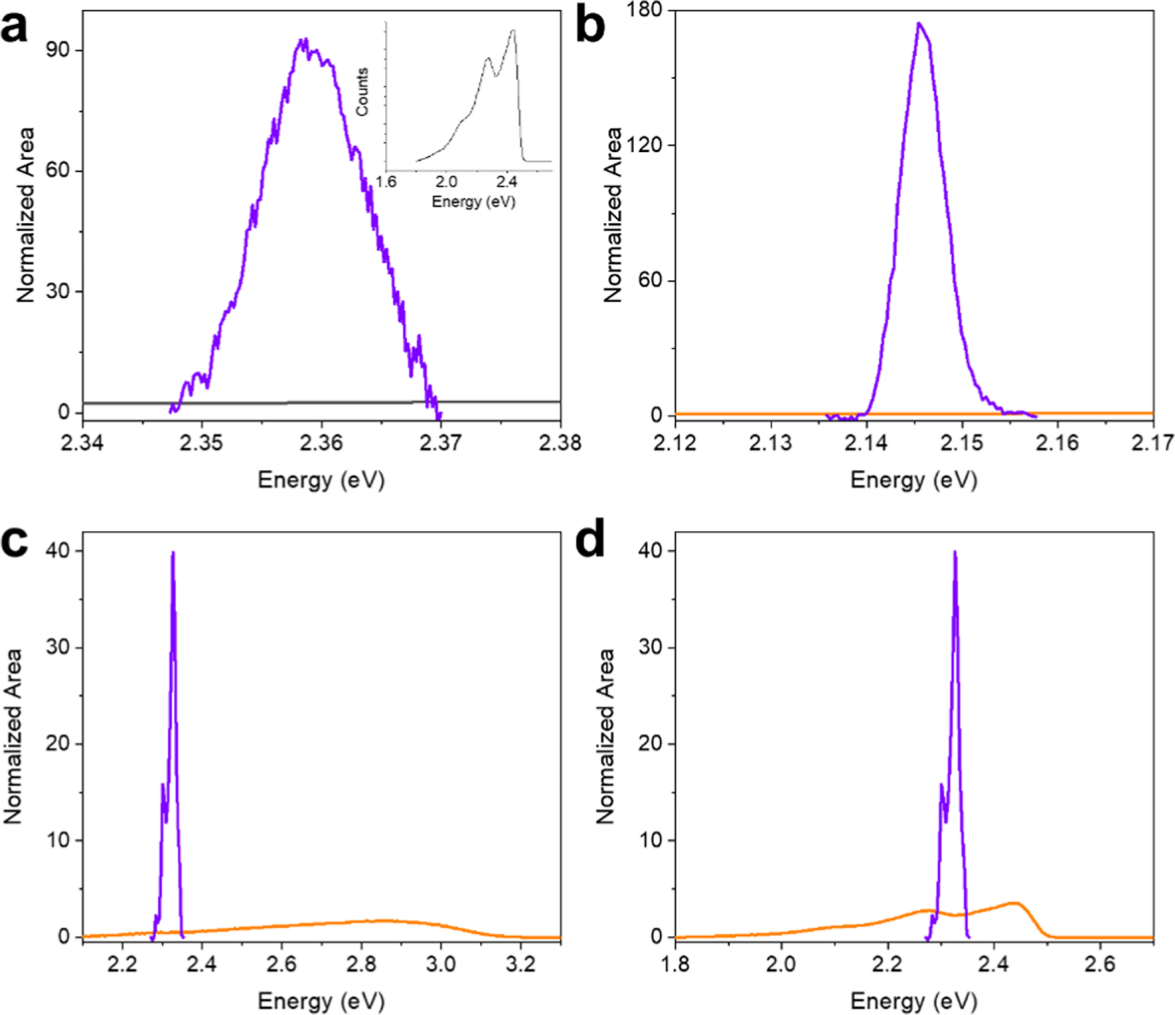
Examples of spectral overlap of antenna emission bands and europium absorption bands, both normalized to unity. Spectra are from this work. (a) **Gd(TTA)**_**3**_**(H**_**2**_**O)**_**2**_ solid-state phosphorescence spectrum (inset) and **EuCl_3_·6H_2_O** aqueous room-temperature absorption spectrum; (b) **Gd(TTA)**_**3**_**(H**_**2**_**O)**_**2**_ solid-state phosphorescence spectrum and 10 μM **Eu(TTA)**_**3**_**(H**_**2**_**O)**_**2**_ in toluene room-temperature emission spectrum. (c) **Gd(TTA)**_**3**_**(H**_**2**_**O)**_**2**_ solid-state singlet emission and **Eu(TTA)**_**3**_**(H**_**2**_**O)**_**2**_ solid-state room-temperature absorption spectrum. (d) **Gd(TTA)**_**3**_**(H**_**2**_**O)**_**2**_ solid-state phosphorescence spectrum and **Eu(TTA)**_**3**_**(H**_**2**_**O)**_**2**_ solid-state room-temperature absorption spectrum.

The spectral overlap integrals were calculated from normalized antenna emission and europium absorption or emission bands. The antenna triplet emission spectra were taken from solid-state **Gd(TTA)**_**3**_**(H**_**2**_**O)**_**2**_ phosphorescence since the energy is expected to be nearer to that of **Eu(TTA)**_**3**_**(H**_**2**_**O)**_**2**_, rather than that of the La^3+^ complex. Singlet emission spectra were taken from **HTTA** and **Gd(TTA)**_**3**_**(H**_**2**_**O)**_**2**_. The Eu^3+^ data were taken from the room-temperature absorption spectra of aqueous **EuCl**_**3**_**·6H**_**2**_**O** and solid-state and toluene-dissolved **Eu(TTA)**_**3**_**(H**_**2**_**O)**_**2**_, except for the ^5^D_0_–^7^F_0_ transition, which was taken from emission spectra. The calculated values do not vary much for different combinations of the above for the same overlap, showing that the assumption is reasonable, and a selection of the resulting graphs is displayed in Figures 4 and S6. In fact, the spectral overlap integrals are all small, in the narrow range of 0.17 to 2.3 eV^–1^ (column 3, Table 1).

### Calculated Energy Transfer Rates: Electric Dipole–Electric Dipole Mechanism

Table 2 summarizes the calculated ET rates using eq 9b. The transfer rates from the antenna triplet state to Eu^3+^ by this mechanism are negligible. The dominant transfer from the singlet state occurs to ^5^D_2_ and ^5^L_6_, although the back-transfer is appreciable for the latter. The maximum total ED–ED transfer rate involving the ^7^F_0_ acceptor state is 5.1 × 10^7^ s^–1^, which is insufficient to quench the singlet emission. The S_1_–T_1_ intersystem crossing rate has been estimated to be typically in the order of 10^8^ s^–1^,^12^ so that this process should dominate ED–ED ET.

**Table 2:** Electric Dipole–Electric Dipole Energy Transfer Rates for **Eu(TTA)**_**3**_**(H**_**2**_**O)**_**2**_^a^

ET transition^b^	*P*_D_ (ED)	*P*_A_ (ED)	SO (eV^–1^)	ν̅_av_ (cm^–1^)	*W*_ET_^f^ (s^–1^)	*W*_ET_^b^ (s^–1^)
T_1_–^5^D_0_	6.53E-9	1.66E-10	1.21	18417	0.001	2.2E-8
		7.9E-8			0.491	1.1E-5
T_1_–^5^D_1_	6.53E-9	7.2E-10	2.29	19291	0.008	7.7E-4
S_1_–^5^D_0_	0.05	1.66E-10	0.197	21357	956	9.9E-15
		7.9E-8			4.6E5	4.7E-12
S_1_–^5^D_1_	0.05	7.2E-10	0.551	22231	1.071E4	1.6E-9
S_1_–^5^D_2_	0.05	1.32E-6	1.335	23481	4.26E7	0.35
		1.36E-8			4.39E5	3.63E-3
S_1_–^5^D_3_	0.05	2.74E-8	1.292	24891	7.62E5	5.1E3
S_1_–^5^L_6_	0.05	1.82E-6	0.172	25278	6.53E6	1.83E6
S_1_–(^7^F_1_–^5^D_1_)	0.05	6.1E-8	0.494	22086	8.24E5	9.7E-9
		8.5E-6			1.15E8	1.4E-6
T_1_–(^7^F_1_–^5^D_1_)	6.53E-9	6.1E-8	2.317	19146	0.67	0.02
		8.5E-6			94	2.8

a *P*_D_ and *P*_A_ are the donor and acceptor ED oscillator strengths. SO is the spectral overlap integral and ν̅_av_ is the donor–acceptor average energy. *W*_ET_^*f*^ and *W*_ET_^*b*^ are, respectively, the forward and backward rates according to the Boltzmann equation. The distance *R* in eq 9b was fixed at 4.5 Å as discussed above.^21^ Only the ED contribution to the oscillator strength of ^7^F_0_ → ^5^D_1_ has been considered. The ED-MD (MD = magnetic dipole) ET rate is zero for a centrosymmetric system^62^ and is expected to be small in other cases.^54^ The oscillator strength of S_1_ → S_0_ is given as 0.05, which is probably 1 order of magnitude too high (SI, Section S3), and if so, the calculated *W*_ET_ then needs to be reduced by a factor of 10. Alternative oscillator strengths are given for ^7^F_0_ → ^5^D_0_ and the hypersensitive ^7^F_0_ → ^5^D_2_ transition, according to the data in Table S4.

b The initial Eu^3+^ state is ^7^F_0_ unless indicated, and the final antenna state is S_0_.

The appreciable population of ^7^F_1_ at room temperature leads to its participation in the ET processes. Two values are listed for the oscillator strength of the ED-allowed transition ^7^F_1_ → ^5^D_1_ in Table 2, taken from Table S4. The spectral overlaps are displayed for the ligand singlet and triplet transitions involving ^7^F_1_ at room temperature in Figure 4c,d, respectively, and the singlet channel is much faster (Table 2) for the ED–ED mechanism. Processes involving ^7^F_1_ → ^5^D_0_ (Figure S6k,l) are not considered here because the ED intensity of this transition is very weak.

### Electric Dipole–Electric Quadrupole (ED–EQ) ET Mechanism

The antenna singlet and triplet transitions may follow orbitally allowed ED transitions. Besides forced ED pure electronic transitions and vibronically allowed ED transitions, some transitions of Ln^3+^ may be EQ allowed. However, the spectral intensity of such transitions is very weak. By contrast, relative to ED–ED ET, the ED–EQ ET mechanism may occur, for *fully allowed* ED and EQ transitions, respectively, with the ratio about (*a*_0_/*R*)^2^,^52,54^ where *a*_0_ is the Bohr radius (5.291772...× 10^–11^ m) and *R* is the donor–acceptor separation, as above. For *R* ∼ 4.5 Å, this ratio is 0.014, so ED–ED ET is more important. However, the 4f^N^ – 4f^N^ transitions of Ln^3+^ are usually not first-order ED-allowed and are rather weaker. Taking the ^7^F_0_ → ^5^D_2_ transition as an example because it is the most intense EQ allowed transition considered herein, eq 14 gives the ratio *P*(ED–EQ)/*P*(ED–ED) of 73 so that the ED–EQ ET mechanism in this case can give a rate up to ∼3.0 × 10^9^ s^–1^. The use of eq 12b (with the oscillator strength of the EQ acceptor transition ^7^F_0_ → ^5^D_2_ (6.7 × 10^–12^)^55^ inserted into eq 11b) gives the result 3.9 × 10^8^ s^–1^, which is similar to the decay rate of S_1_ → S_0_.

In summary, the combined ED–EQ and ED–ED rates from the S_1_ state can therefore account for the quenching of singlet emission. The ET contributions from the triplet state to Eu^3+^ by the above mechanisms are very small, leading to its common description of “Dexter ET”.

### Rate Equation Approach

**Figure 5:**
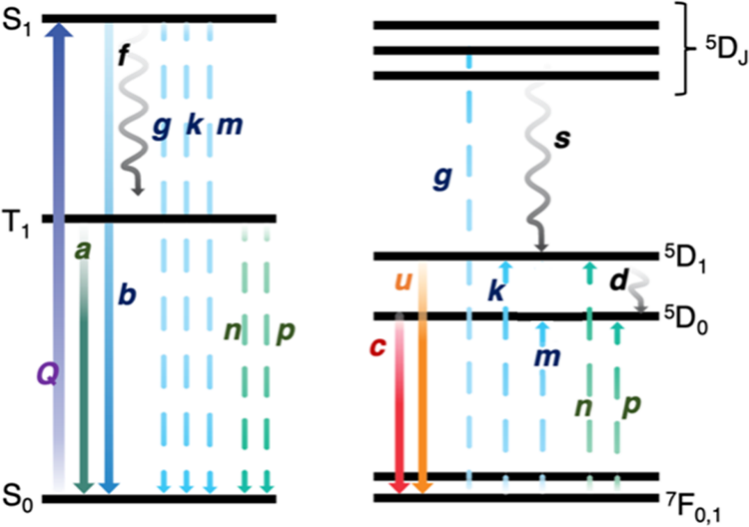
Rate constants in the independent systems model.

We apply an independent systems model to calculate the energy transfer rate to Eu^3+^ in **Eu(TTA)**_**3**_**(H**_**2**_**O)**_**2**_. This means that the overall metal–ligand transitions are considered independently and concurrently so that sensible ground-state populations are conserved. Seven levels are included. The antenna levels are S_1_, T_1_, and S_0_. Other levels such as T*_n_* were found to be of minor importance here. The Eu^3+^ levels are labeled DJ that include ^5^L_6_, ^5^D_3_, and ^5^D_2_ combined; ^5^D_1_, ^5^D_0_, and ^7^F_0_. The latter comprises ^7^F_0_ and ^7^F_1_ for simplicity here, but we have also performed calculations using separate levels. However, we have taken into account that the occupations of ^7^F_0_ and ^7^F_1_ are about 0.6 and 0.4 at room temperature, but 1.0 and 0.0, respectively, at 10 K. The rate constants are listed in Table 3. From the above temperature dependence of ^5^D_0_ lifetime, we are confident that a charge transfer state is not involved in the energy transfer processes. The relevant experimental data for **Eu(TTA)**_**3**_**(H**_**2**_**O)**_**2**_ have been previously published (Figure 5).^8^

**Table 3:** Rate Constants, Their Meanings, and Values Adopted in the Calculations

rate constant^a^	meaning	value 300 K	value 10 K
*Q*	S_0_ → S_1_ excitation	10	10
*a*	T_1_ → S_0_ nonradiative and radiative decay	1E6	1E2
*b*	S_1_ → S_0_ fluorescence decay	3E8	3E8
*f*	S_1_ → T_1_ intersystem crossing	1E9	1E9
*c*	^5^D_0_ → ^7^F_J_ luminescence	5000	3906
*g*	S_1_ → S_0_^7^F_0_ → D_J_ energy transfer	1E9	1E9
*k*	S_1_ → S_0_^7^F_0_ → ^5^D_1_ energy transfer	1.1E8	1.1E4
*m*	S_1_ → S_0_^7^F_0_ → ^5^D_0_ energy transfer	1.17E6	4.6E5
*n*	T_1_ → S_0_^7^F_0_ → ^5^D_1_ energy transfer	3.33E7	2.84E7
*p*	T_1_ → S_0_ F_0_ → ^5^D_0_ energy transfer	1.5E7	1.5E7
*s*	DJ → ^5^D_1_ internal conversion	1E8	1E8
*d*	^5^D_1_ → ^5^D_0_ internal conversion	2.5E6	2.5E5
*u*	^5^D_1_ → ^7^F_J_ luminescence	1E4	1.5E5

a Unit s^–1^ or mol^–1^ s^–1^.

The rate equations considered are as follows:
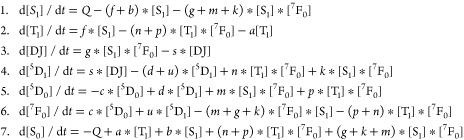


These equations were solved in Maple 2021^51^ by the Rosenbrock method with the initial concentrations of [S_0_] = 1, [^7^F_0_] = 1, and others zero. The concentrations at 5 ns were determined, and these were employed in the further calculation with zero excitation (*Q* = 0). The rationale for parameter values is given in SI, Section S7. The values are representative, and we have not made an effort to fine-tune them to simulate experimental data.

The first calculations assumed, as often done, that the energy transfer to Eu^3+^ arises solely from the triplet state. In this case, the rate parameters *k*, *m*, and *g* were set to zero. The 300 K risetime of ^5^D_1_ from Figure 4 in ref (8) is ∼30 ns, which infers an ET rate (parameter n) of 3.33 × 10^7^ s^–1^ if the transfer is only from T_1_. The 300 K decay time of ^5^D_1_ is 0.4 μs.^8^ Using the other illustrative values in Table 3, for this zero singlet ET scenario (Szero), the calculated concentrations of the different states are plotted against time in Figure S7 (black lines) and subsequently fitted by mono- or biexponential functions (red lines). The calculated risetime of ^5^D_1_ was found to be 20 ns, which is the same as the triplet decay time. To simulate 10 K conditions, the calculation was repeated with the contributions to energy transfer from ^7^F_1_ omitted, Figure S8 (Table 3, column 4, also with the ^5^D_1_ and ^5^D_0_ lifetimes adjusted for their temperature dependence). Again, the ^5^D_1_ risetime equals the triplet decay time, but it only increases to 23 ns, far from the experimental value ∼70 ns as in Figure 4b, ref (8). We therefore consider that other parameters would need to change to achieve a longer risetime but reducing the intersystem crossing rate (*f*) by a factor of 10 from 300 to 10 K has little effect upon the ^5^D_1_ risetime (Figure S9) and increasing parameter *a* to 10^6^ s^–1^ has no effect (Figure S10).

An alternative model involves ET from solely the singlet state (the Tzero model), as displayed in Figure S11 for 300 K. Again, there is a ^5^D_1_ risetime in the ns range, in this case 10 ns. By comparison with the previous Szero model, this leads to the conclusion that the experimental observation of a ns risetime for ^5^D_1_ does not enable one to judge if the ET route involves singlet or triplet ET, let alone the operating mechanism, as has often been done. Taking into account the changes in ^5^D_0_ and ^5^D_1_ lifetimes and the absence of ET involving ^7^F_1_, the 10 K profiles are shown in Figure S12 and the ^5^D_1_ risetime does not differ from that at room temperature. However, if the internal conversion rate (parameter s) is reduced by a factor of 10 as in Figure S13, which is reasonable for such a temperature change, the ^5^D_1_ risetime increases up to 102 ns. This could be a signature of mainly singlet energy transfer.

The two above models, Szero and Tzero, show that the feeder state, T_1_ or ^5^D_2_, determines the ^5^D_1_ risetime and that while, from 300 to 10 K, a 10-fold change of ^5^D_2_–^5^D_1_ internal conversion rate is reasonable and produces a longer risetime, the change of rate involving T_1_ only slightly modifies the risetime. Hence, in principle, we can distinguish singlet or triplet ET by monitoring the change in ^5^D_1_ risetime with temperature.

**Figure 6:**
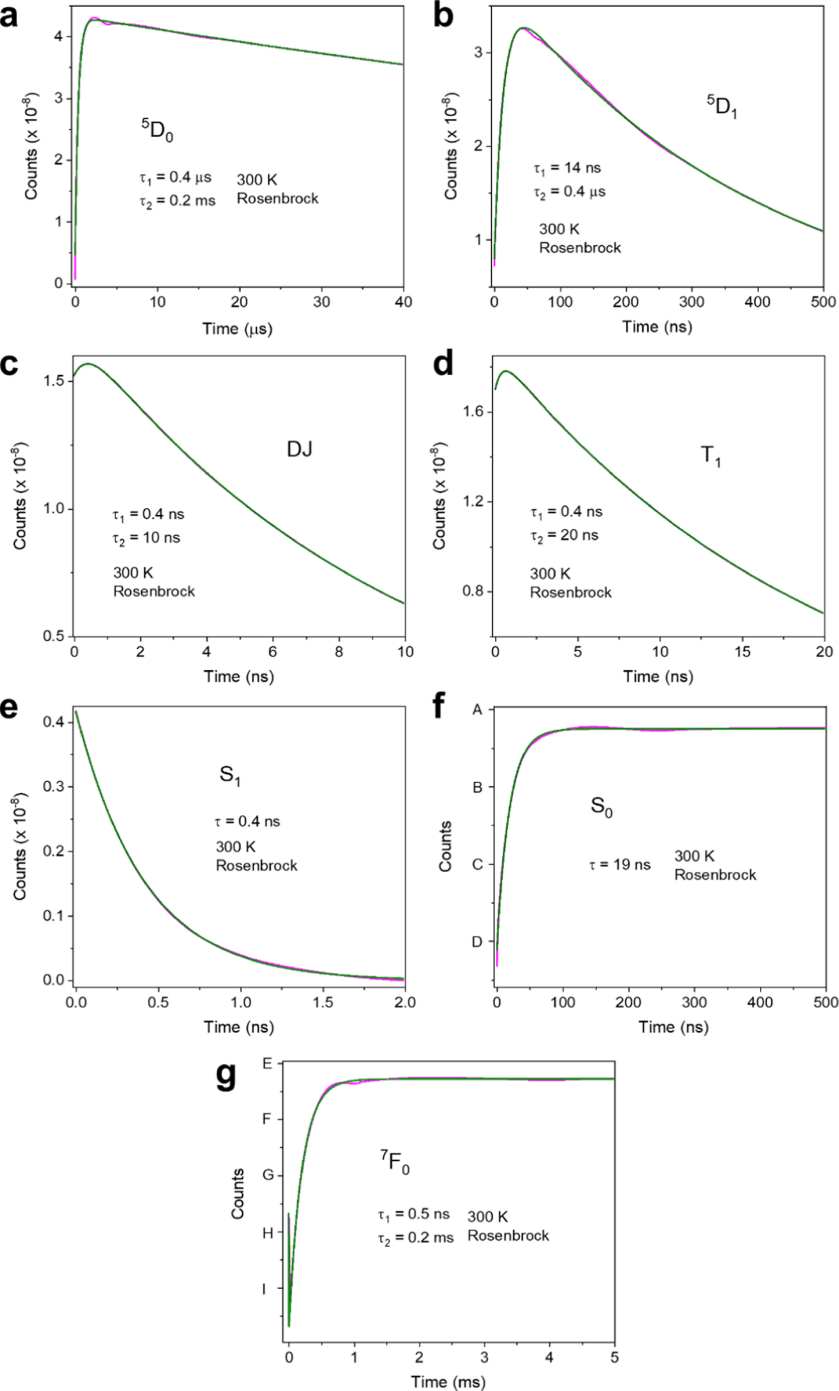
Calculated (black lines) 300 K time profiles of levels using the Rosenbrock method, following a 5 ns pulse. (a) ^5^D_0_; (b) ^5^D_1_; (c) DJ; (d) T_1_; (e) S_1_; (f) S_0_; (g) ^7^F_0_. The parameters are in columns 3 and 7 of Table 3. The red lines are fits to the calculated data using mono- or biexponential functions, with the fitted lifetimes as indicated. In S_0_ and F_0_: A 1.000000002; B 0.9999999948; C 0.999999988; D 0.9999999811; E 1.000000003; F 0.999999993; G 0.9999999835; H 0.9999999739; I 0.9999999643.

Figure 6 displays the ET scenario from ligand to Eu^3+^ at 300 K with combined singlet and triplet energy transfer as in columns 3 and 7, Table 3: 96% from S_1_ and 4% from T_1_. Remember that the exchange contribution to T_1_ is not known, but we can put an upper limit on it from the ^5^D_1_ risetime.

The ^5^D_1_ risetime is intermediate (14 ns) between the Szero and Tzero models. The profile for ^7^F_0_ is interesting because it displays both the nanosecond decrease (as in Szero) and the long risetime as in Tzero. The latter is determined by ^5^D_0_ decay, whereas the former is the same as the T_1_ decay time and the S_0_ risetime. Monitoring the population of ^7^F_0_ (although difficult) may therefore be fruitful when considering energy transfer mechanisms.

**Figure 7:**
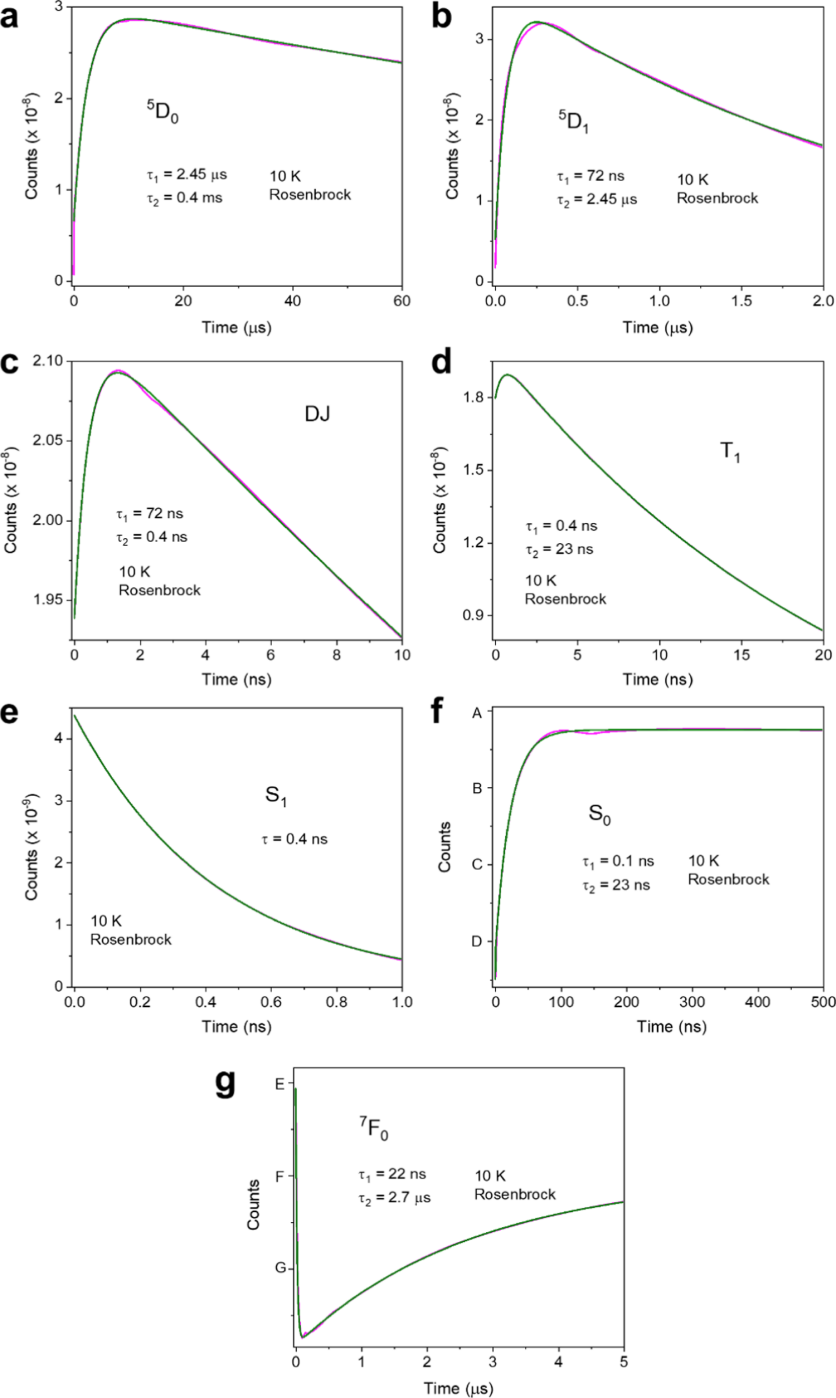
Calculated 10 K time profiles of levels using the Rosenbrock method, following a 5 ns pulse (black lines). (a) ^5^D_0_; (b) ^5^D_1_; (c) DJ; (d) T_1_; (e) S_1_; (f) S_0_; (g) ^7^F_0_. The parameters are in columns 4 and 8 of Table 3, with the exception that *s* = 10^7^ s^–1^. The red lines are fits to the calculated data using mono- or biexponential functions, with the fitted lifetimes as indicated. In S_0_ and F_0_: A 1.000000002; B 0.9999999948; C 0.999999988; D 0.9999999811; E 0.9999999788; F 0.9999999708; G 0.9999999629.

Using the parameters in Table 3, columns 4 and 8, to simulate the 10 K kinetics (Figure S14), the ^5^D_1_ risetime is intermediate between the decay times of ^5^D_2_ (DJ) and T_1_ but is similar to the room temperature value. However, taking into account the slowing of the ^5^D_2_–^5^D_1_ internal conversion rate by an order of magnitude from 300 to 10 K (with rate constant s changed in Table 3, column 8, to 10^7^ s^–1^, Figure 7), the ^5^D_1_ risetime becomes much longer.

We summarize the results from the model as follows:

1. The maximum total ET rate from the ligand singlet state to the acceptor ^7^F_0_ state *via* the ED–ED mechanism is of the order 10^7^ s^–1^. The rate involving the acceptor state ^7^F_1_ is up to the order 10^8^ s^–1^. The intensity of Eu^3+^ emission decreases with increasing temperature despite an increasing ET rate involving the ^7^F_1_ acceptor state. The maximum rate for ED–EQ transfer (10^8^–10^9^ s^–1^) is sufficient to quench the singlet emission. We are presently unable to calculate the ET rate for exchange interaction but our calculations of the ^5^D_1_ risetime indicate a value in the range of 10^7^ s^–1^.
2. The ET rate from the triplet state by ED–ED and ED–EQ mechanisms is very weak but is still enough to quench the long lifetime triplet emission.
3. The observation of a nanosecond risetime for ^5^D_1_ does not enable distinction of triplet or singlet ET, let alone the mechanism. The ^5^D_1_ risetime may be contributed by several processes.
4. Following the ^5^D_1_ risetime as a function of temperature and using the temporal profile of ^7^F_0_ may provide useful information concerning the energy transfer route.

## Conclusions

We have employed the Dexter formalism^54^ to investigate the ET mechanisms and rates from an antenna to a lanthanide ion, with the complex **Eu(TTA)**_**3**_**(H**_**2**_**O)**_**2**_ as a case study. This compound may not have been a good choice in view of its rapid decomposition in moist air, but our spectra are consistent with those of previous studies. Considerable differences exist between the spectra for the complex in the solid state and in dilute solution. These arise from the enormous concentration factor in the solid state and merit further study. The use of the experimental data for oscillator strengths and spectral overlap enables ET rates to be calculated for ED–ED and ED–EQ ET. As previously shown,^61^ the Förster and Dexter formalisms are the same for ED–ED transfer, with the exception of local field effects. The independent systems model is successful in reproducing the temporal variation of experimental data. The model can be applied on different timescales and is general for Eu^3+^ complexes except for special cases, such as the involvement of a charge transfer state, where the model can be further adapted by its inclusion. This study does show that time-gated spectroscopy together with transient absorption measurements could provide detailed information to understand the kinetics of energy transfer in Eu^3+^ complexes.

In the Supporting Information, we have commented upon the software LUMPAC and JOYSpectra, which both use the model of Malta. We consider that the use of an approximation for the exchange integral and a fixed value may not be justified. The major outstanding problem therefore lies with the calculation of exchange ET rate. Some previous reports have considered the evaluation of two-center exchange integrals, and we will study these to apply the formalism of Dexter and calculate the exchange contribution to ET.

## Supporting Information Available

Experimental (S1); computation of molecular structure (S2); singlet energy and oscillator strength (S3); oscillator strengths (S4); spectral overlap integral (S5); spectral overlap diagrams (S6); rate equation model (S7); calculations using the model of Malta (S8); units and conversions used in this work (S9); and references (S10) (PDF)
