# Supplementary material for: Energy Transfer Mechanism and Quantitative Modeling of Rate from an Antenna to a Lanthanide Ion
Source: J Phys Chem A. 2022 Oct 6;126(41):7418–31. doi: 10.1021/acs.jpca.2c03965 (PMC9589723; doi:10.1021/acs.jpca.2c03965)
Supplement: Supplementary file 1 — jp2c03965_si_001.pdf [file jp2c03965_si_001.pdf]

SUPPORTING INFORMATION

Energy Transfer Mechanism and Quantitative Modeling of Rate from an Antenna to a Lanthanide Ion

Peter A. Tanner,†,* Waygen Thor,† Yonghong Zhang,‡ Ka-Leung Wong†

†Department of Chemistry, Hong Kong Baptist University, Waterloo Road, Kowloon Tong, Hong Kong S.A.R., P.R. China

‡State Key Laboratory of Chemistry and Utilization of Carbon Based Energy Resources, Key Laboratory of Oil and Gas Fine Chemicals, Ministry of Education & Xinjiang Uygur Autonomous Region, Urumqi Key Laboratory of Green Catalysis and Synthesis Technology, College of Chemistry, Xinjiang University, Urumqi 830017, Xinjiang, P.R. China

CONTENTS

Item	Title	Page,S
**S1**	**EXPERIMENTAL**	2
Table S1	Reported bands in the infrared spectrum of $Eu(TTA)_3(H_2O)_2$	2-3
Figure S1	Thermogravimetric analysis results for $Eu(TTA)_3(H_2O)_2$	3
**S2**	**COMPUTATION OF MOLECULAR STRUCTURE**	4
Table S2	Bond distances in the first coordination sphere of Eu in $Eu(TTA)_3(H_2O)_2$	4
**S3**	**SINGLET ENERGY AND OSCILLATOR STRENGTH**	4
Figure S2	(a) Room temperature emission spectra of **HTTA** and $\mathbf{Eu(TTA)_3(H_2O)_2}$ in the solid state (b) 77 K emission spectra of $\mathbf{La(TTA)_3(H_2O)_2}$ (blue) and **HTTA** (magenta); excitation spectra of 550 nm (green) and 580 nm (red) emission of solid $\mathbf{La(TTA)_3(H_2O)_2}$. (c) Concentration dependence of room temperature absorption spectrum of $\mathbf{Eu(TTA)_3(H_2O)_2}$ in toluene. (d) Singlet emission decay of solid **HTTA** at 77 K. (e) Room temperature excitation spectrum of (red) 610 nm emission of solid $\mathbf{Eu(TTA)_3(H_2O)_2}$; (magenta) 77 K excitation spectrum of 613 nm emission of 10 μM $\mathbf{Eu(TTA)_3(H_2O)_2}$ in toluene. (f) Singlet emission decay of solid $\mathbf{La(TTA)_3(H_2O)_2}$ at 77 K. (g) Calculated CASSCF-NEVPT2 absorption spectra of $\mathbf{Eu(TTA)_3(H_2O)_2}$ in the gas phase using different active spaces.	6
Table S3	Selected europium energy levels in materials	7
**S4**	**OSCILLATOR STRENGTHS**	7
Figure S3	Room temperature absorption spectrum of $EuCl_3{\cdot}6H_2O$ dissolved in water	8
Table S4	Measured oscillator strengths of Eu^{3+} transitions for various systems	8-9
**S5**	**THE SPECTRAL OVERLAP INTEGRAL**	9
Figure S4	Overlap of two Gaussians	10
Figure S5	Spectral overlap of area under two Gaussians	11
**S6**	**SPECTRAL OVERLAP DIAGRAMS**	12
Figure S6	Spectral overlap diagrams	12-13
**S7**	**RATE EQUATION MODEL**	13
Figure S7	Zero singlet ET. 300 K energy level time profiles following a 5 ns pulse with the parameters in Table 3, columns 3 and 7, except with k, m and g set equal to zero	14
Figure S8	Zero singlet ET, 10 K. This calculation uses the same parameters as in Table 3 columns 4 and 8, with the triplet state energy transfer only involving $^7F_0$, not $^7F_1$	15
Figure S9	Zero singlet ET, 10 K. This calculation uses the same parameters as in Table 3	15

	columns 4 and 8, and Figure S8, except f = 10^8 s^{-1}	
Figure S10	Zero singlet ET, 10 K. This calculation uses the same parameters as in Table 3 column 4 and Figure S8, except f = 10^8 s^{-1}, a = 10^6 s^{-1}	15
Figure S11	Zero Triplet ET. 300 K energy level time profiles with the parameters in Table 3, columns 3 and 7, except with n and p set equal to zero	16-17
Figure S12	Zero Triplet ET. 10 K energy level time profiles with the parameters in Table 3, columns 4 and 8, except with n and p set equal to zero	18
Figure S13	Zero Triplet ET. 10 K energy level time profiles with the parameters in Table 3, columns 4 and 8, except with n and p set equal to zero and s = 1E7 s^{-1}	19
Figure S14	Calculated 10 K time profiles of levels using the Rosenbrock method, following a 5 ns pulse. The parameters are in columns 4 and 8 of Table 3 (black lines).	20
**S8**	**CALCULATIONS USING THE MODEL OF MALTA**	21
Table S5	Calculation of spectral overlap integral using direct integration with Maple 2021 and with Eq. S4 for *F*	21-22
Table S6	Comparison of results for ET rates of $Eu(TTA)_3(H_2O)_2$ using the software LUMPAC and JOYSpectra	23-24
Table S7	Calculated squared reduced spin matrix elements	25
**S9**	**UNITS AND CONVERSIONS USED IN THIS WORK**	28
**S10**	**REFERENCES**	29

S1. EXPERIMENTAL

Wavenumber (cm^{-1})			
Ref. S1	Ref. S2	This work	Calc. DFT
1592	1588	1583	1590
1508	1514	1514	1471
1499	--	1505	
1397	1412	1409	1406
1346	1359	1353	1352
1289	1305	1285	1281
1245	1253	1249	1260
1220	1232	1231	1223
1196	1196	--	
1179	--	1182	1187
1134	1144	1131	
1072	1084	1082	1098
1054	1064	1061	1063

1033		1036	
1028		--	
1004		1012	
927		933	957
921		--	892
856		860	827
789		787	792
782		--	775
762		769	
746		749	
729		726	735
707		715	706
689		694	
677		681	

Table S1. Reported bands in the infrared spectrum of **Eu(TTA)$_3$(H$_2$O)$_2$**. Ref. S1: a list of bands is given with wavelengths in microns; Ref. S2: scanned from the spectrum in the Supporting Information of Ref. S2. Calc. DFT: gas phase, MWB52 PBE0/D3BJ/def2-TZVP level.

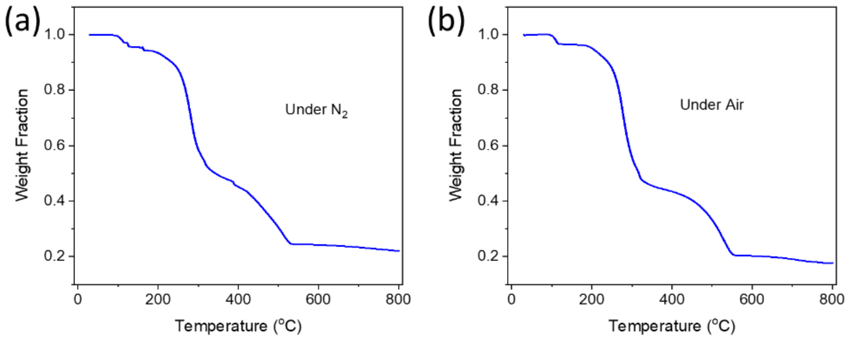

Figure S1. Thermogravimetric analysis results for **Eu(TTA)$_3$(H$_2$O)$_2$** using atmospheres of (a) nitrogen and (b) air. The initial loss of mass commences at 100 °C, up to 120-130 °C, and corresponds to the loss of two water molecules. The X-Ray diffractogram of **Eu(TTA)$_3$(H$_2$O)$_2$** is given in Ref. S3. Attempts in the present study to employ X-ray diffraction led to sample decomposition.

S2. COMPUTATION OF MOLECULAR STRUCTURE

Atom	Distance (Å)				
	DFT[a] Gas	DFT[b] solution	DFT[c] solution	Triclinic Structure[S4]	Monoclinic Structure[S3]
O8(w)	2.499	2.542	2.506	2.424	2.507
O7(w)	2.619	2.612	2.586	2.408	2.545
O6	2.384	2.424	2.366	2.419	2.466
O5	2.358	2.363	2.397	2.405	2.384
O4	2.386	2.394	2.421	2.330	2.496
O3	2.368	2.402	2.369	2.380	2.381
O2	2.396	2.371	2.445	2.369	2.388
O1	2.379	2.393	2.377	2.389	2.507

Table S2. Bond distances in the first coordination sphere of Eu in **Eu(TTA)$_3$(H$_2$O)$_2$**.

[a]MWB52-PBE0/D3BJ/def2-TZVP; [b]including CPCM(toluene); [c]Ref. S4, $CHCl_3$, BP86/def2-TZVP.

S3. SINGLET ENERGY AND OSCILLATOR STRENGTH

We envisage that after ultraviolet excitation, rapid intersystem crossing occurs from the higher singlet states to the lowest one, which then can undergo intersystem crossing and/or direct ET to Eu^{3+}. The transfer occurs from the $S_1 – S_0$ zero phonon line (ZPL). This energy may be calculated by the ΔSCF method and is generally at several thousand cm^{-1} lower than that calculated for the corresponding vertical transition by TD-DFT.[S5] The room temperature spectra emission spectra of **HTTA**, **La(TTA)$_3$(H$_2$O)$_2$** and **Eu(TTA)$_3$(H$_2$O)$_2$** are too broad for estimation of the ZPL energy, Figure S2(a), but it is readily assigned at 25410 cm^{-1} at 77 K, Figure S2(b).

The room temperature absorption spectra of **Eu(TTA)$_3$(H$_2$O)$_2$** in toluene at different concentrations are displayed in Figure S2(c) and are similar to the literature spectra. The singlet oscillator strength can be calculated from absorption, Eq. (2-6) or emission, Eq. (1) measurements. The absorption spectrum of **Eu(TTA)$_3$(H$_2$O)$_2$** in acetonitrile has been reported[S1,S2,S6] and our deconvolution of the first broad absorption band in Ref. S7 into two Gaussians gives the oscillator strength of 0.05 for the lower energy one, using Eq. 5. However, our calculations show that there are not two, but about 10 different singlet states in this spectral region and fitting our experimental spectra with multiple Gaussians is not accurate. The calculated TD-DFT oscillator strengths of the lowest singlet state of **Eu(TTA)$_3$(H$_2$O)$_2$** in toluene

(vertical transition energy 24871 cm^{-1}, 402 nm) and in the gas phase (vertical transition energy 29867 cm^{-1}, 335 nm) are 0.0048 and 0.0083, respectively.

Figure S2e shows the room temperature excitation spectra of **Eu(TTA)$_3$(H$_2$O)$_2$** in toluene and in the solid state. The singlet absorption band is red-shifted and broadened for the solid, where Eu^{3+} transitions are observed. The packing of **Eu(TTA)$_3$(H$_2$O)$_2$** units increases by a magnitude of $2.2x10^5$ from 10 μM concentration in a solvent to the solid and this has been taken to indicate greater spin-orbit coupling and hence faster intersystem crossing to the triplet state.[S8] The appearance of the europium absorption bands indicates greater competition of internal Eu^{3+} excitation with the antenna ET in the solid state, and may involve $4f^6 - 4f^6$ cross-relaxation processes in the solid state. The measured ratio of the integrated areas ($^7F_0 \rightarrow {}^5D_2$)/($^7F_0 \rightarrow {}^5D_1$) [at, in cm^{-1}: 21552/19052] in the excitation spectrum of solid **Eu(TTA)$_3$(H$_2$O)$_2$** at room temperature in Figure S2e is 110, which is far from the nearly equal ratios from the absorption spectra. This may indicate that $^5D_0$ is more favorably populated in the solid state by excitation into $^5D_2$, rather than $^5D_1$, due to the cross-relaxation: $^5D_2 + {}^7F_0 \rightarrow {}^5D_0 + {}^7F_5$. There is a first-order *J*-selection rule prohibiting $^5D_1 \rightarrow {}^5D_0$ vibrational relaxation, mentioned below.

The singlet emission of **Eu(TTA)$_3$(H$_2$O)$_2$** is totally quenched at room temperature when it is dissolved in toluene, but a very weak band with maximum at ~425 nm is observed for the solid (Figure S2a). This could be due to dissociation, but if it corresponds to **Eu(TTA)$_3$(H$_2$O)$_2$** singlet emission, then the singlet ET/intersystem crossing rate is of the same order as the singlet lifetime for **Eu(TTA)$_3$(H$_2$O)$_2$** in the solid state at room temperature. The singlet emission of **HTTA**, with maximum at 441 nm, exhibits monoexponential decay with the lifetime of 1.3 ns at room temperature and 5.1 ns at 77 K (Figure S2d). The corresponding lifetime for **La(TTA)$_3$(H$_2$O)$_2$** at 77 K is 3.5±0.3 ns (Figure S2f). From Eq. 1, a 5 ns radiative lifetime is equivalent to an oscillator strength of about 0.19, whereas a 20 ns radiative lifetime would give P_{if} ~ 0.05. In the case of **La(TTA)$_3$(H$_2$O)$_2$**, a 20 ns radiative lifetime would represent a quantum efficiency of 18%.

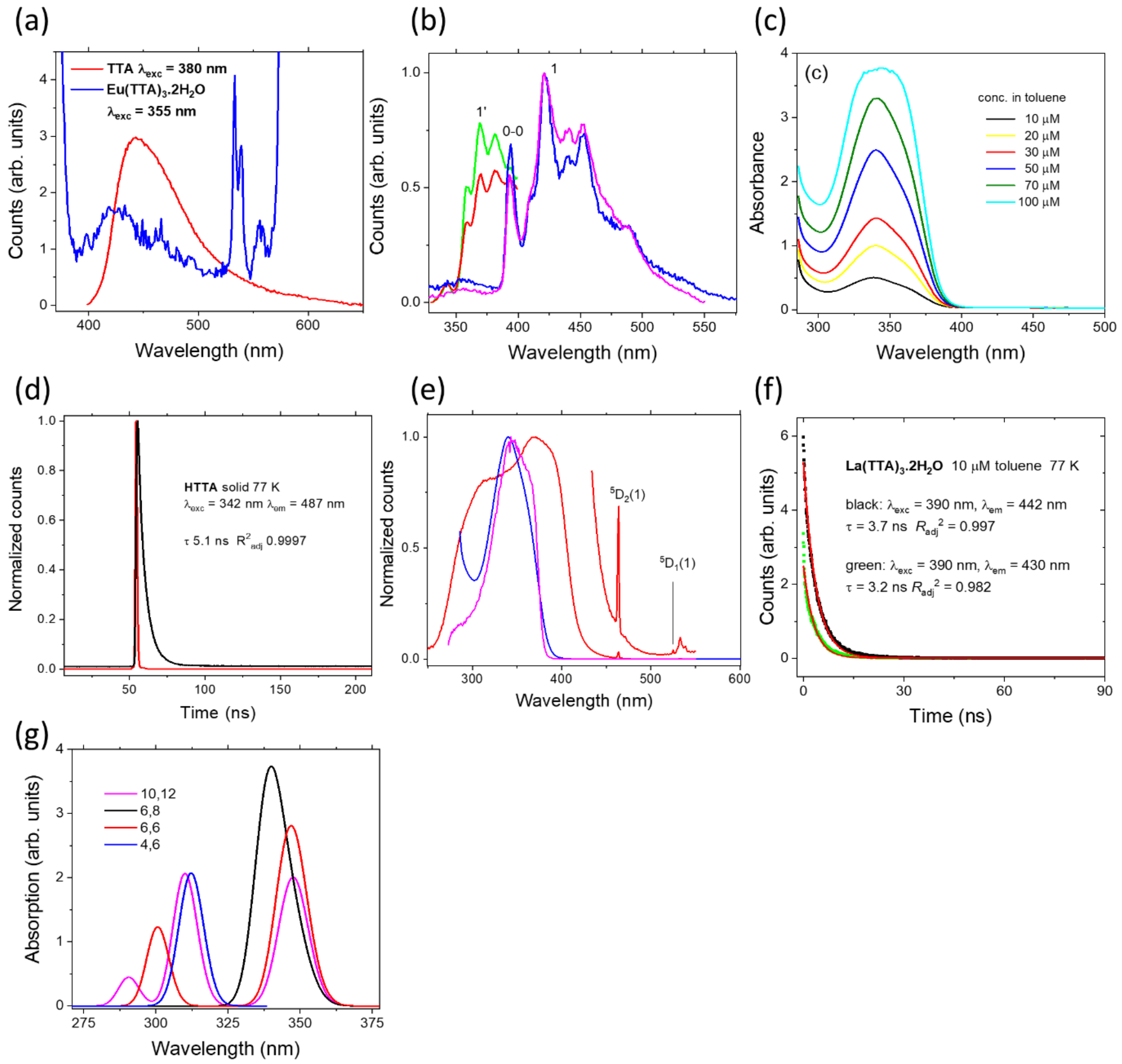

Figure S2. (a) Room temperature emission spectra of **HTTA** and **Eu(TTA)$_3$(H$_2$O)$_2$** in the solid state using the excitation wavelengths indicated (b) 77 K emission spectra of **La(TTA)$_3$(H$_2$O)$_2$** (blue) and **HTTA** (magenta) at 10 μM concentration in toluene using 300 nm excitation; excitation spectra of 550 nm (green) and 580 nm (red) emission of solid **La(TTA)$_3$(H$_2$O)$_2$**. The $S_1 – S_0$ zero phonon line is indicated as 0 - 0 with the most prominent vibronic transitions involving the C=O stretching vibration of 1660±20 cm^{-1} marked as 1 and 1'. (c) Concentration dependence of room temperature absorption spectrum of **Eu(TTA)$_3$(H$_2$O)$_2$** in toluene. (d) Singlet emission decay of solid **HTTA** at 77 K. The laser prompt is shown. (e) Room temperature excitation spectrum of (red) 610 nm emission of solid **Eu(TTA)$_3$(H$_2$O)$_2$**. The inset shows an ordinate scale expansion; (blue) Room temperature excitation spectrum of 613 nm emission of 50 μM **Eu(TTA)$_3$(H$_2$O)$_2$** in toluene; (magenta) 77 K excitation spectrum of 613 nm emission of 10 μM **Eu(TTA)$_3$(H$_2$O)$_2$** in toluene. (f) Singlet emission decay of solid **La(TTA)$_3$(H$_2$O)$_2$** at 77 K. (g) Calculated CASSCF-NEVPT2 absorption spectra of **Eu(TTA)$_3$(H$_2$O)$_2$** in the gas phase using different active spaces, as

indicated. The spectra show bands in the region of 310 nm and 340-350 nm but the relative intensity changes with choice of active space.

Energy Level	**Y_2O_3:Eu**[S9] bc	**$EuCl_3.6H_2O$**[S10] Energy range (no. of bands)	**$Cs_2NaEuCl_6$**[S11] Energy range (no. of bands)	**LaF_3:Eu**[S12] bc	**$Eu(TTA)_3(H_2O)_2$**
$^5D_0$	17216	17248 (1)	17208 (1)	17293	17303
$^5D_1$	18959	18993-19018 (3)	18961 (1)	19027	19052
$^5D_2$	21421	21446-21514 (5)	21385-21495 (2)	21483	21552
$^5D_3$	24265	24537-(24399) (7)	24261-24370 (3)	24355	
$^5L_6$	24950	24988-25327 (13)	24761-25345 (6)	25325	25146
$^5D_4$, $^5L_9$		27585-27690 (9)			
$^5L_7$			25893-26099 (4)	26357	
$^5G_2$			26162-26175 (2)	26392	
$^5G_3$			26216 (1)	26622	
$^5L_7$			26240 (1)		
$^5G_3$			26338 (1)		
$^5G_4$			26339 (1)	26735	
$^5L_7$			26345 (1)		
$^5G_4$			26424 (1)		

Table S3. Selected europium energy levels in materials. Values are given in cm^{-1}. bc barycenter. The Eu^{3+} states above 26000 cm^{-1} are of much more mixed parentage.

S4. OSCILLATOR STRENGTHS

Some values of oscillator strength for Eu^{3+} transitions are listed in Table S4, taken from the literature and the present study.

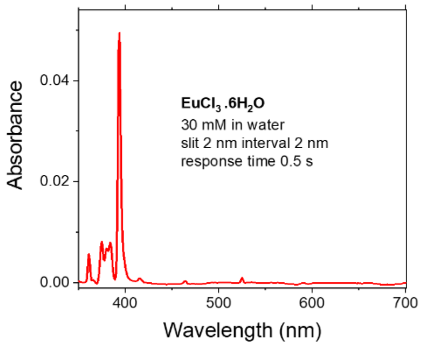

Figure S3. Room temperature absorption spectrum of **$EuCl_3 \cdot 6H_2O$** dissolved in water.

System	State	Transition	Eq	Osc. Str. Abs.	Osc. Str. Corr.[a]	Ref.	Comments
**$EuCl_3$**	aq./band max						
	17241	$^7F_0 \rightarrow {}^5D_0$	5	1.66E-10	3.19E-10		
	19047	$^7F_0 \rightarrow {}^5D_1$	5	1.41E-8	2.71E-8		
	21562	$^7F_0 \rightarrow {}^5D_2$	5	1.36E-8	2.61E-8		
	24038	$^7F_0 \rightarrow {}^5D_3$	5	2.74E-8	5.27E-8		
	25380	$^7F_0 \rightarrow {}^5L_6$	5	1.82E-6	3.50E-6		
	26031	$^7F_0 \rightarrow {}^5G_2$	5	3.07E-7	5.90E-7		
	16949	$^7F_1 \rightarrow {}^5D_0$	5	8.15E-9	1.70E-8		
**$EuCl_3 \cdot 6H_2O$**	Solid/bands						
	17260	$^7F_0 \rightarrow {}^5D_0$		2.2E-9		S13	ED
	19003-19028	$^7F_0 \rightarrow {}^5D_1$		1.25E-8		S13	Mainly MD; 7.2E-10 ED
	21456-21522	$^7F_0 \rightarrow {}^5D_2$		1.44E-8		S13	ED
**$Eu(TTA)_3(H_2O)_2$**	Toluene						

		$^7F_0 \rightarrow {}^5D_2$	5	1.32E-6	2.54E-6		
**Eu(DBM)(H_2O)**	Solid	$^7F_0 \rightarrow {}^5D_0$		2.6E-8<br>7.9E-8		S14	
		$^7F_0 \rightarrow {}^5D_1$		1.2E-7		S14	
		$^7F_0 \rightarrow {}^5D_2$		3.91E-6		S14	
		$^7F_1 \rightarrow {}^5D_0$	8	2.4E-7		S15	
		$^7F_1 \rightarrow {}^5D_1$		(8.5±0.3) E-6		S14	Mean of absorption and emission
**$Na_3[Eu(ODA)_3]\cdot 2NaClO_4\cdot 6H_2O$**	Solid	$^7F_1 \rightarrow {}^5D_1$		6.1E-8		S16	Mainly ED
		$^7F_1 \rightarrow {}^5D_0$	8	2.85E-7		S15	
		$^7F_0 \rightarrow {}^5D_1$	8	3.18E-8		S16	

Table S4. Measured oscillator strengths of Eu^{3+} transitions for various systems. Data from the present work or given references. DBM dibenzylmethanido. ODA oxydiacetato. [a]The values are corrected for the population of the $^7F_0$ and $^7F_1$ states, assuming a barycenter of 380 cm^{-1}.

The intensity of these bands can arise from several mechanisms. The $^7F_0$ - $^5D_1$ and $^7F_1$ – $^5D_0$ transitions are mainly of magnetic dipole (MD) character. The $^7F_0$ – $^5D_0$ transition is forbidden under Judd-Ofelt theory, but the breakdown of the closure approximation, *J*-mixing and the Wybourne-Downer process[S17] enable this transition to gain intensity. Furthermore, it can gain intensity *via* crystal strain to give coincident defect site bands. We measured the oscillator strength for this transition of Eu^{3+} in aqueous **$EuCl_3$** (Figure S3) to be $1.7x10^{-10}$, whereas Hellwege and Kahle[S13] gave the magnitude of $2.2x10^{-9}$ and Ferrier et al.[S17] gave the value $1.3x10^{-8}$ for **Y_2SiO_5:Eu^{3+}**. The strongest Eu^{3+} transition in our region of interest is $^7F_0$ – $^5L_6$, with the oscillator strength of the order 10^{-6}, which is mainly of ED character. Electric dipole transitions gain intensity by both static and dynamic coupling mechanisms, in addition to the cross-terms of these.[S14] The ED intensities depend upon the system geometry, charges and dipolar polarizabilities.

S5. THE SPECTRAL OVERLAP INTEGRAL

This integral is of the form:

$$SOI = \int_{-\infty}^{\infty} f_D(E) f_A(E) dE \qquad \text{(S1)}$$

where the $f(E)$ are the normalized donor emission and acceptor absorption spectra on an energy scale. In our case, the antenna donor (D) has a broad emission spectrum, and the lanthanide acceptor (A) spectrum is a sharp band.

For an example, we model these by two Gaussian functions, y(D) and y(A), where y is given by:

$$y = y_0 + \left[A' exp\left\{\frac{-4ln2(E-E_c)^2}{w^2}\right\}\right] / \left[w\sqrt{\frac{\pi}{4ln2}}\right] \qquad \text{(S2)}$$

Here, y_0 is the lower y value; A' is the peak area; E_c is the peak center (unit eV); w = FWHM;

The two Gaussian functions each have $A' = 1$, $y_0 = 0$:

For D, simulating singlet emission, $H = 0.6$ eV, $E_c = 2.77$ eV, and for A, simulating $^5D_0$ absorption, $H = 0.006$ eV, $E_c = 2.14$ eV. Each simulation in Origin employed 20000 data points. Then:

D := 1.566300*exp(-7.707470*(E - 2.770000)^2)

A := 156.632000*exp(-77074.70*(E - 2.140000)^2)

From Maple 2021:[S18]
$\int_{-\infty}^{\infty} f(D)f(A)\, dE = 0.073669\ eV^{-1}$

The integral gives the same result as long as the interval extends over the acceptor profile. Plotting the curves gives Figure S4:

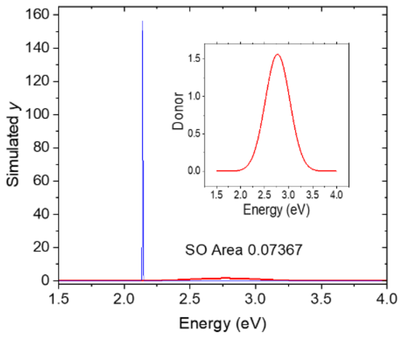

Figure S4. Overlap of two Gaussians. The singlet donor emission is shown in the inset on an expanded ordinate scale.

where the donor is shown on an expanded ordinate scale in the inset. The spectral overlap area was calculated from the columns y(D) and y(A) in Origin 9©,[S19] with rows having the same abscissa E:

area = sum{y(D)[i]*y(A)[i]*(E[i+1] - E[i])} (S3)

and gave the result indicated in the graph, very close to the calculated integral. On the other hand, if we just consider the region where the curves overlap, we have Figure S5, where the area is 47 times smaller.

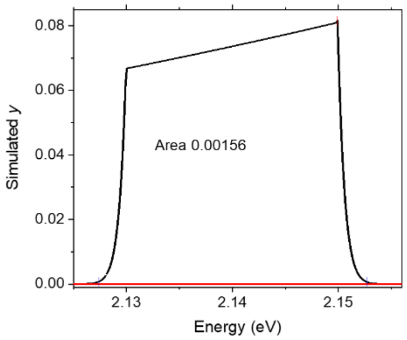

Figure S5. Spectral overlap of area under two Gaussians.

S6. SPECTRAL OVERLAP DIAGRAMS

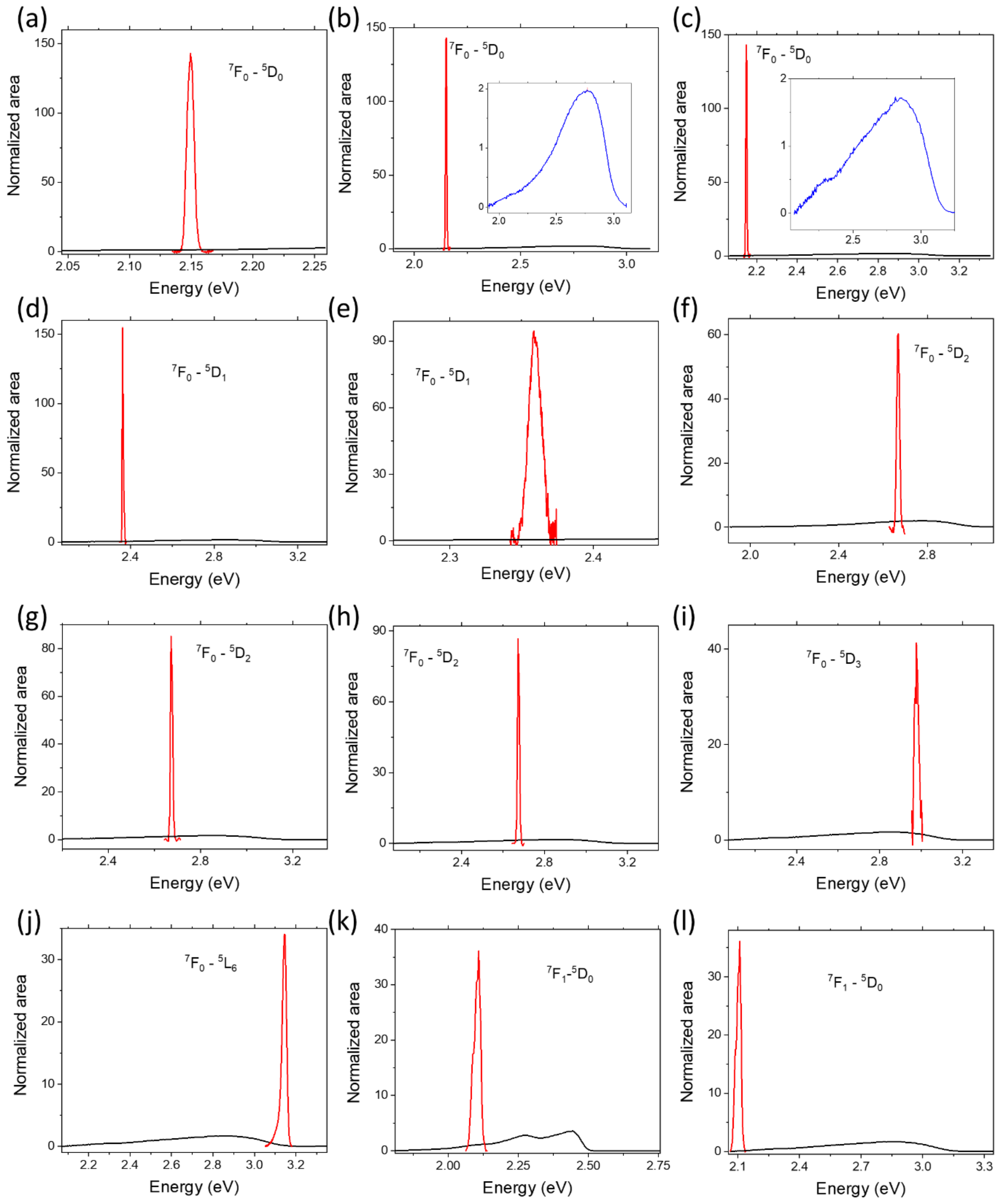

Figure S6. Spectral overlap diagrams. (a) **$Gd(TTA)_3(H_2O)_2$** solid-state phosphorescence $T_1 \rightarrow S_0$ and **$Eu(TTA)_3(H_2O)_2$** solid state 355 nm excited emission spectrum at room temperature. (b) Singlet emission of solid **HTTA** and **$Eu(TTA)_3(H_2O)_2$** solid state 355 nm excited emission spectrum at room temperature. (c) Singlet emission of solid **$Gd(TTA)_3(H_2O)_2$** and emission spectrum of solid **$Eu(TTA)_3(H_2O)_2$** at room

temperature. (d) Singlet emission of solid **Gd(TTA)$_3$(H$_2$O)$_2$** and absorption spectrum of solid **Eu(TTA)$_3$(H$_2$O)$_2$**. (e),(f) Singlet emission of solid **HTTA** and absorption spectrum of aqueous **EuCl$_3$·6H$_2$O**. (g) Singlet emission of solid **Gd(TTA)$_3$(H$_2$O)$_2$** and excitation spectrum of solid **Eu(TTA)$_3$(H$_2$O)$_2$** at room temperature. (h) Singlet emission of solid **Gd(TTA)$_3$(H$_2$O)$_2$** and absorption spectrum of solid **Eu(TTA)$_3$(H$_2$O)$_2$**. (i), (j) Singlet emission of solid **Gd(TTA)$_3$(H$_2$O)$_2$** and absorption spectrum of aqueous **EuCl$_3$·6H$_2$O**. (k) Triplet emission of solid **Gd(TTA)$_3$(H$_2$O)$_2$** and emission spectrum of 10 μM **Eu(TTA)$_3$(H$_2$O)$_2$** in toluene. (l) Singlet emission of solid **Gd(TTA)$_3$(H$_2$O)$_2$** and emission spectrum of 10 μM **Eu(TTA)$_3$(H$_2$O)$_2$** in toluene. In k, l, the populations of $^7F_1$ crystal field states have been adjusted to change from emission to absorption.

S7. RATE EQUATION MODEL

Initial parameter values. The value of Q is arbitrary[S20] and its variation within a reasonable range does not affect the conclusions herein. Phosphorescence is quenched at room temperature. The value of parameter b indicates a fluorescence lifetime of 3.3 ns. The intersystem crossing rate (f) in lanthanide complexes is generally taken to be in the range of 10^8-10^9 s^{-1}. The larger value is taken because the fluorescence is (mostly?) quenched. We take the room temperature value[S21] of 0.2 ms for the $^5D_0$ lifetime, and the value 0.256 ms at 10 K (Fig. 3a, Ref. S21). The singlet ET rate is the sum of calculated values for $S_1 \rightarrow$ DJ (g), $^5D_1$ (k) or $^5D_0$ (m) with $^7F_0$, $^7F_1$ at 300 K, and only for $^7F_0$ at 10 K. The parameter values for g, k, m are similar to those calculated in Table 3 of the manuscript. The triplet ET rate is the sum of $T_1 \rightarrow {}^5D_1$ (n) or $^5D_0$ (p) with $^7F_0$, $^7F_1$ at 300 K, and only for $^7F_0$ at 10 K. The $^5D_1$ risetime at 300 K or 10 K in Fig. 4b of Ref. S21 was used to calculate rate constant n, considering the level occupations, overlap integrals and degeneracies of $^7F_0$, $^7F_1$. Also, we have employed the trial values of n = 1E8 s^{-1} and 1E9 s^{-1}, even reducing g = 1E8 s^{-1}, but then the $^5D_1$ risetimes are too fast. This puts an upper limit to the exchange contribution to parameter n. The parameter value p is not known but is expected to be smaller than n. The internal conversion parameter s was initially set at 10^8 s^{-1}. The gap Eu^{3+} ($^5D_2$ – $^5D_1$) is 2500 cm^{-1}, which can be spanned by two phonons. However, the most effective nonradiative process cannot just be estimated from the energy gap and the maximum phonon frequency.[S22] The energy gap for Pr^{3+} ($^3P_0$ - $^1D_2$) is 2600-2700 in **M_2O_3:Pr^{3+}** (M = Y, Lu) with the room temperature multiphonon relaxation rate >10^7 s^{-1}.[S23] Gaps between the energy levels of **$LiYF_4$:Nd^{3+}** of 938-1649 cm^{-1} are associated with nonradiative decay rates of 10^8-10^9 s^{-1}.[S22] The parameters d and u were adjusted to give the $^5D_1$ lifetimes at 300 K and 10 K. The fact that the risetime of $^5D_0$ equals the lifetime of $^5D_1$ (Fig. 5, Ref. S21) means that the radiative lifetime of $^5D_1$ is orders of magnitude longer than the nonradiative relaxation rate. The $^5D_1$ – $^5D_0$ nonradiative decay is a special case where $J = 1 \rightarrow J = 0$ selection rule limits the nonradiative relaxation rate, just as for Pr^{3+} ($^3P_1$ – $^3P_0$) with the gap of 621 cm^{-1} but with the slow rate of 3.33×10^5 s^{-1}.[S24] The change in nonradiative relaxation rate of ($^5D_1$ – $^5D_0$) from 10 K to 300 K is estimated to be an order of magnitude.[S25]

Zero energy transfer scenarios at 300 K and 10 K. The zero singlet energy transfer at 300 K gives the following results using a 5 ns pulse.

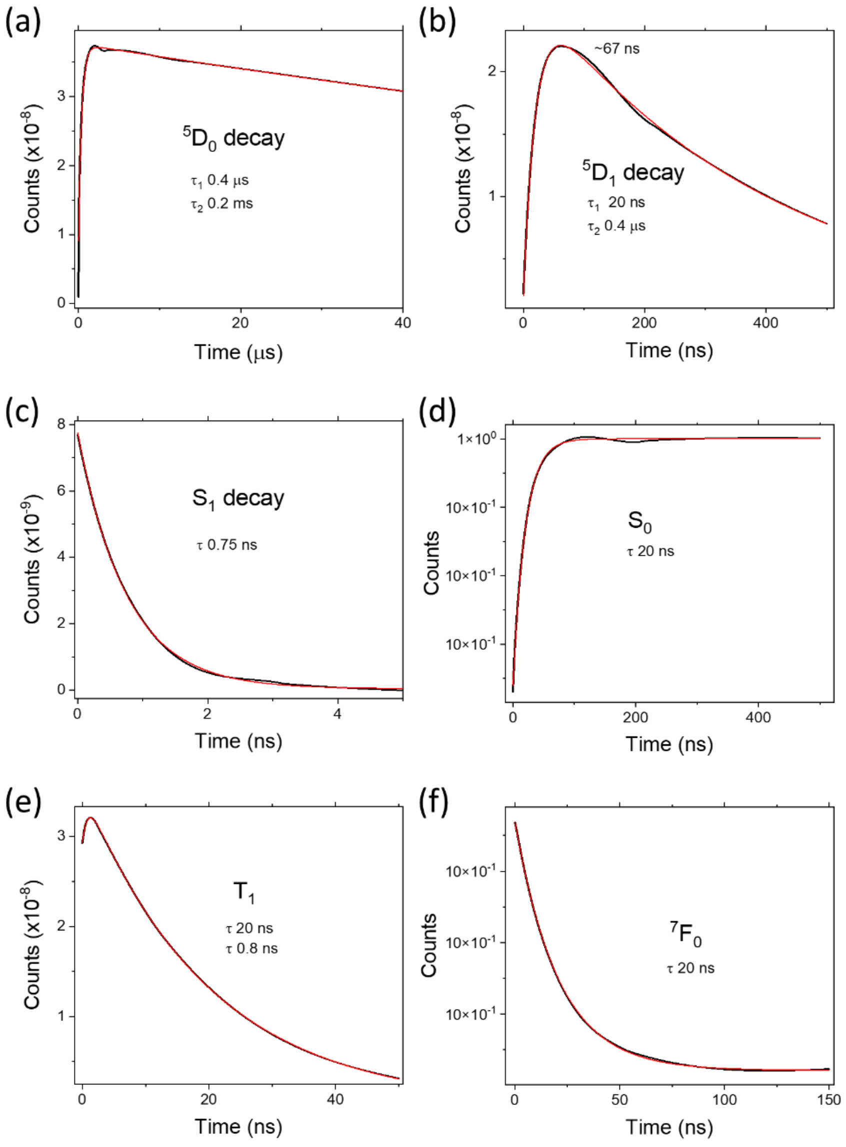

Figure S7. Zero singlet ET. 300 K energy level time profiles following a 5 ns pulse with the parameters in Table 3, columns 3 and 7, except with k, m and g set equal to zero. (a) $^5D_0$; (b) $^5D_1$; (c) S_1; (d) S_0; (e) T_1; (f) F_0. The population of DJ is zero. Black curves are calculated by Maple 2021 and red curves are fitted by Origin 9 mono- or bi-exponential functions, with lifetimes as indicated. In these and in the following figures, the Counts for F_0 and S_0 are 0.99999999 or similar.

Zero singlet energy transfer scenario at 10 K. The results are plotted in Figure S8.

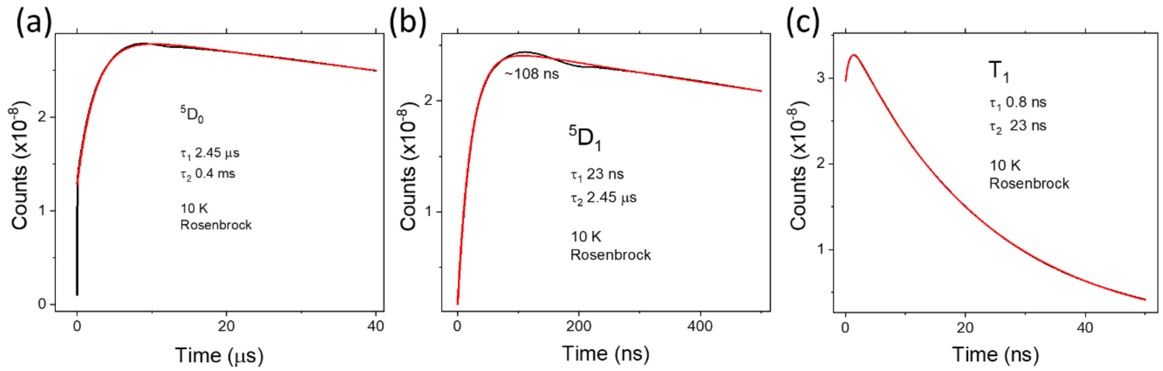

Figure S8. Zero singlet ET, 10 K. This calculation uses the same parameters as in Table 3 columns 4 and 8, with the triplet state energy transfer only involving $^7F_0$, not $^7F_1$. (a) $^5D_0$; (b) $^5D_1$; (c) T_1. Parameters k, m and g are set equal to zero. Again, a 5 ns pulse was used.

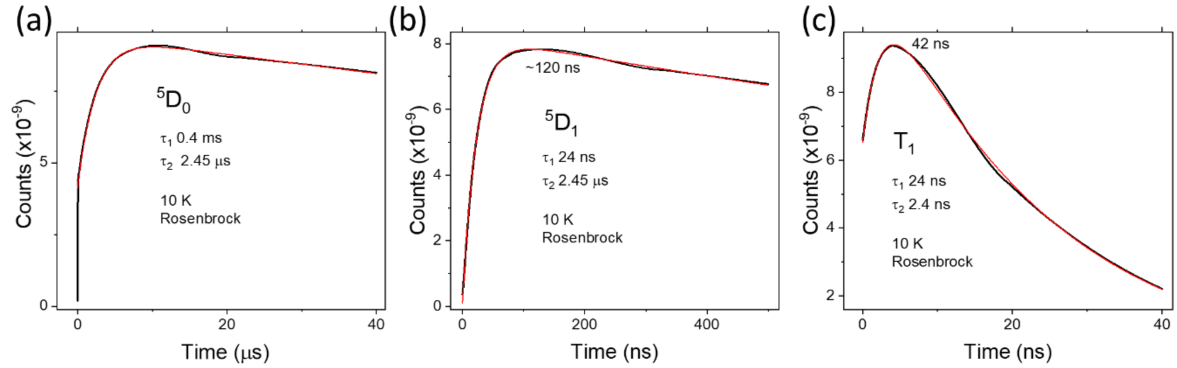

Figure S9. Zero singlet ET, 10 K. This calculation uses the same parameters as in Table 3 columns 4 and 8, and Figure S8, except f = 10^8 s^{-1}. As above a 5 ns pulse was used. (a) $^5D_0$; (b) $^5D_1$; (c) T_1. The triplet state energy transfer only involves $^7F_0$, not $^7F_1$. Black curves are calculated by Maple 2021 and red curves are fitted by Origin 9 mono- or bi-exponential functions, with lifetimes as indicated.

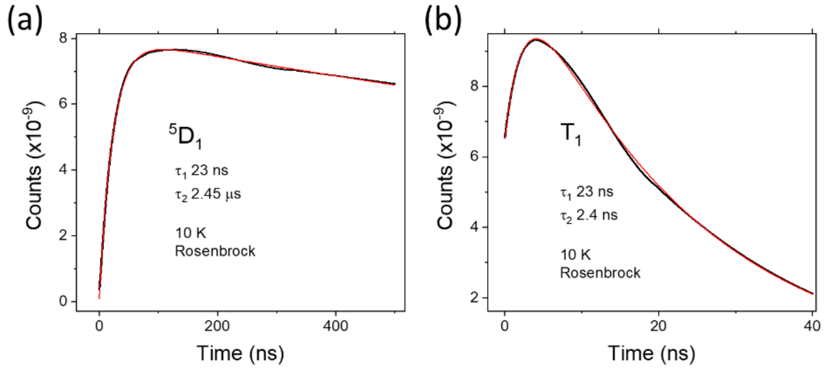

Figure S10. Zero singlet ET, 10 K. (a) $^5D_1$; (b) T_1. This calculation uses the same parameters as in Table 3 column 4 and Figure S8, except f = 10^8 s^{-1}, a = 10^6 s^{-1}. As in Figures S8 and S9, parameters k, m and g are set equal to zero. A 5 ns pulse was used. The triplet state energy transfer only involves $^7F_0$, not $^7F_1$. Black

curves are calculated by Maple 2021 and red curves are fitted by Origin 9 mono- or bi-exponential functions, with lifetimes as indicated.

Zero triplet energy transfer scenario, 300 K and 10 K

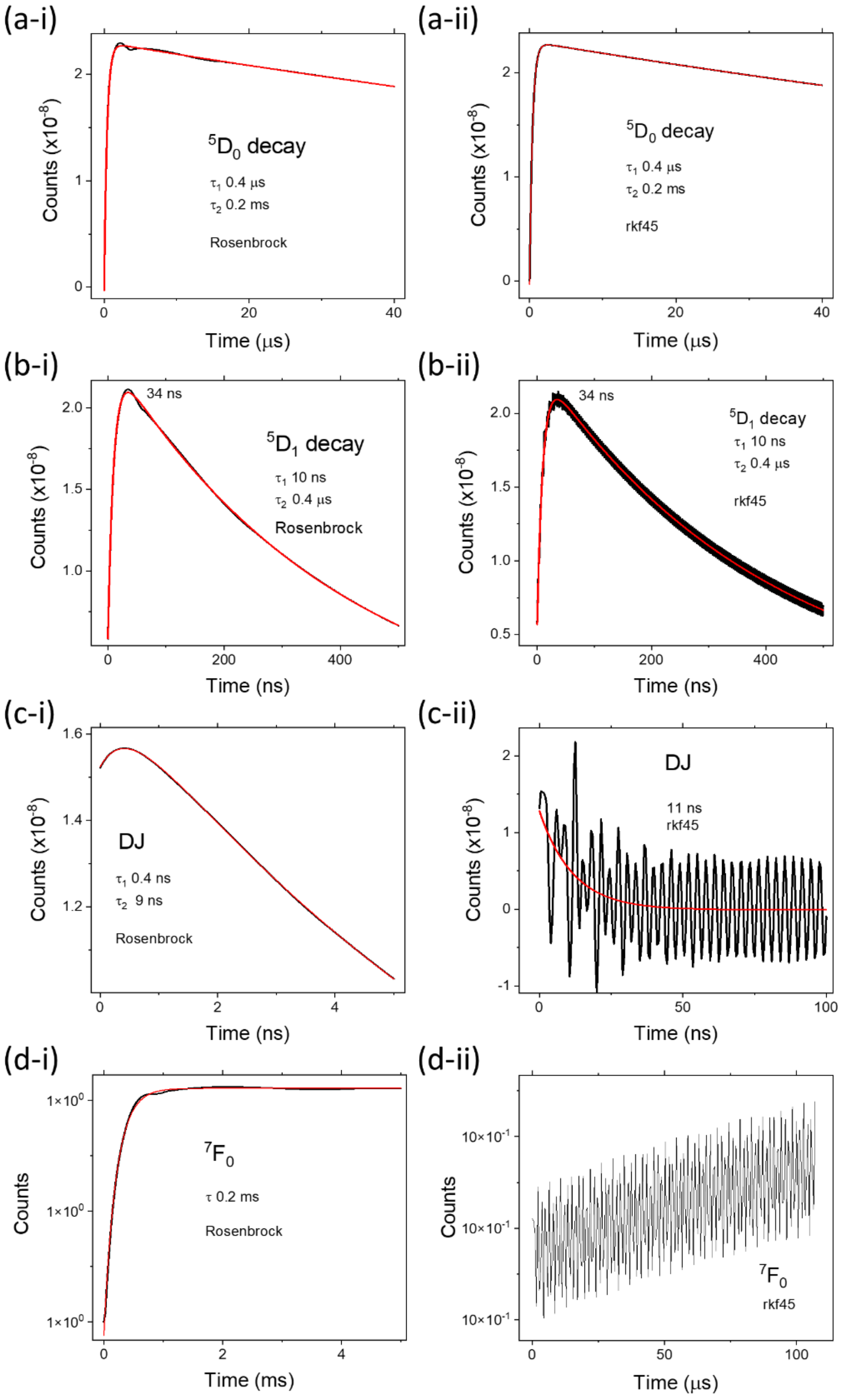

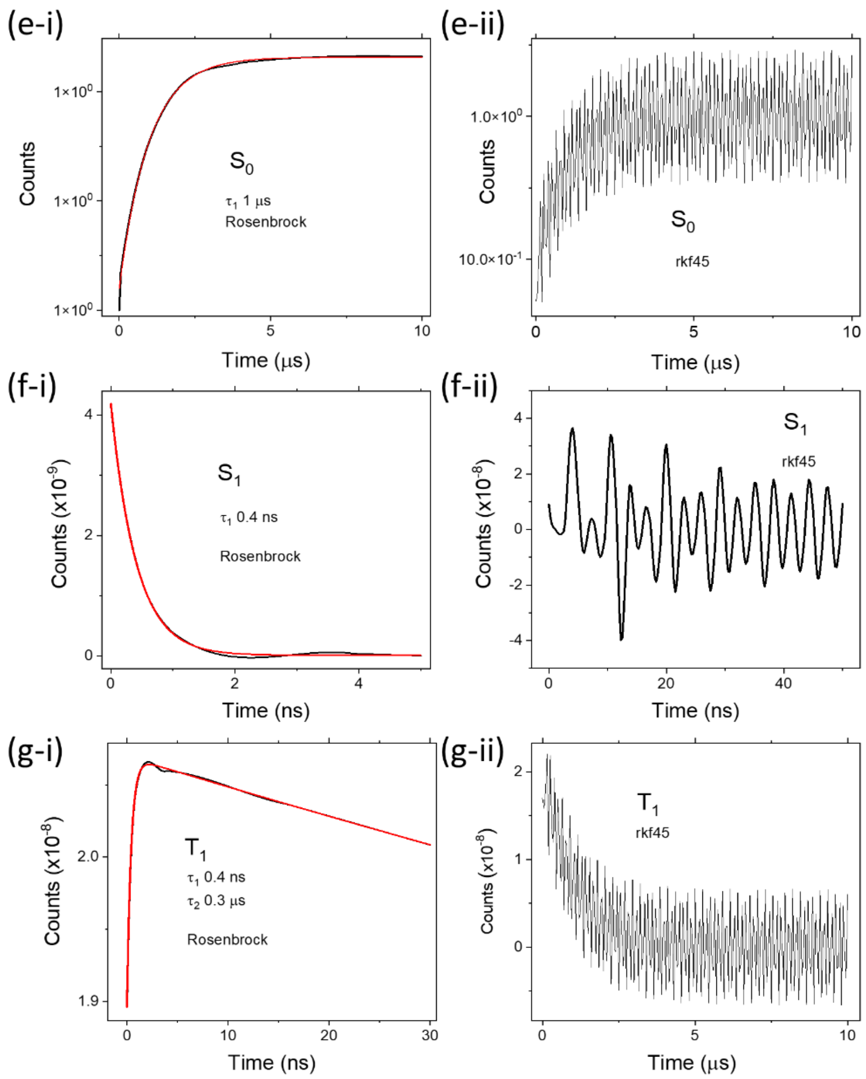

Figure S11. Zero Triplet ET. 300 K energy level time profiles with the parameters in Table 3, columns 3 and 7, except with n and p set equal to zero. A 5 ns pulse was used. (a) $^5D_0$; (b) $^5D_1$; (c) DJ; (d) $^7F_0$; (e) S_0; (f) S_1; (g) T_1. Black curves are calculated by Maple 2021 and red curves fitted by Origin mono- or bi-exponential functions with lifetimes as indicated. The differential equations were solved by the Rosenbrock (labeled (i)) or Runge-Kutta-Fehlberg (rkf45) (labeled (ii)) algorithm with automatic error estimation using rules of order 4 and 5, as indicated. The Rosenbrock method is more stable but gives an artifact (for example at 3.5 μs for $^5D_0$) which is not present in the rkf45 method.

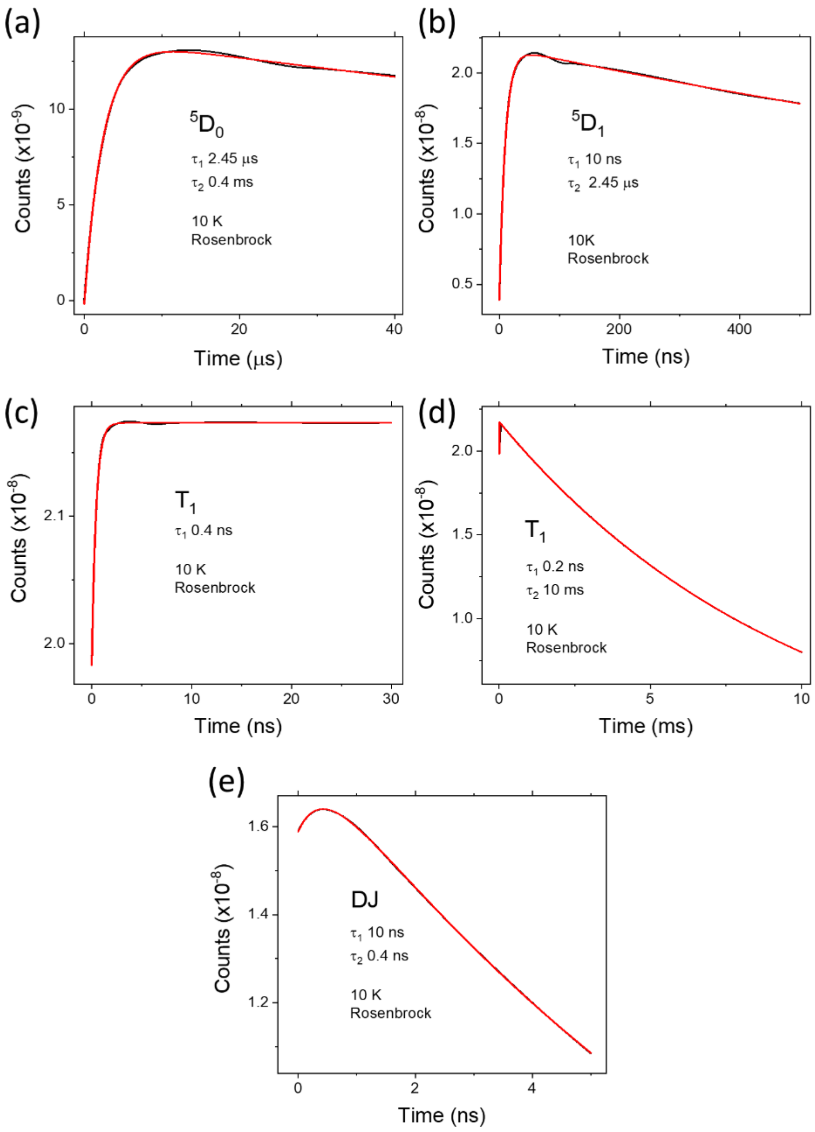

Figure S12. Zero Triplet ET. 10 K energy level time profiles with the parameters in Table 3, columns 4 and 8, except with n and p set equal to zero. A 5 ns pulse was used. (a) $^5D_0$; (b) $^5D_1$; (c) T_1, ns range; (d) T_1, ms range; (e) DJ. Note the different ranges for T_1 in (c), (d). Black curves are calculated by Maple 2021 and red curves are fitted by Origin 9 mono- or bi-exponential functions, with lifetimes as indicated.

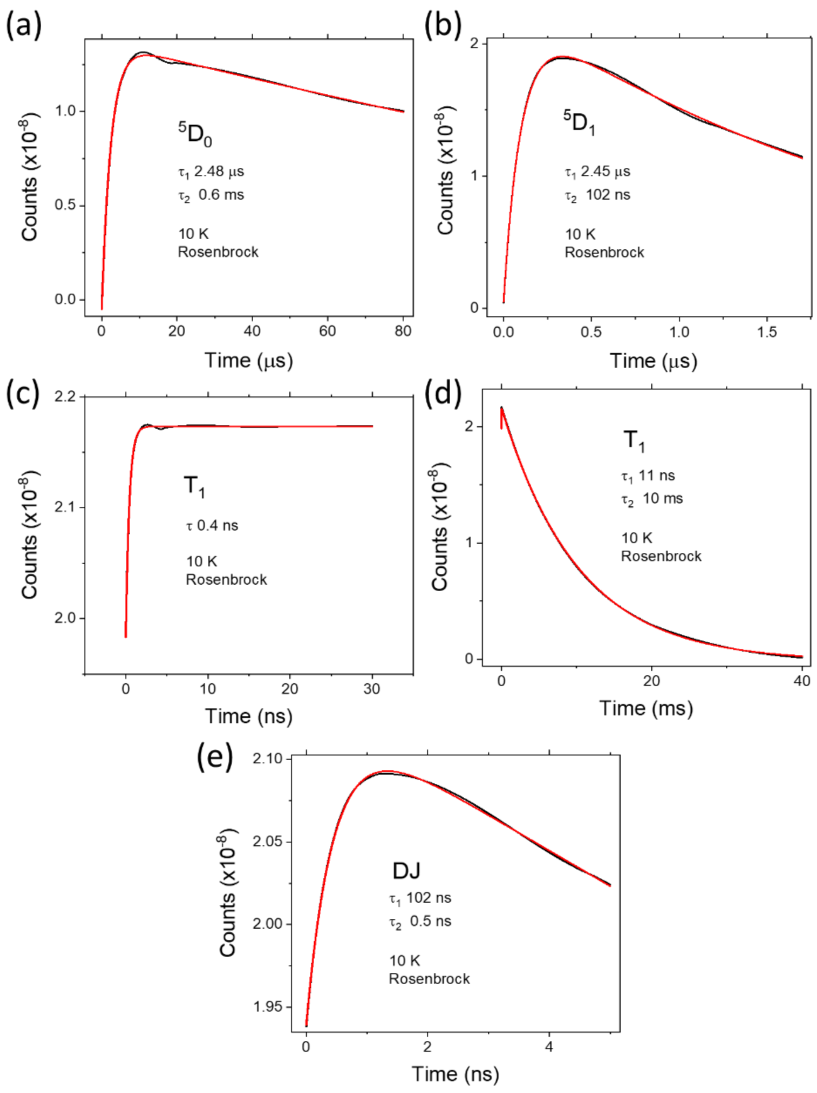

Figure S13. Zero Triplet ET. 10 K energy level time profiles with the parameters in Table 3, columns 4 and 8, except with n and p set equal to zero and s = 1E7 s^{-1}. A 5 ns pulse was used. (a) $^5D_0$; (b) $^5D_1$; (c) T_1, ns range; (d) T_1, ms range; (e) DJ. Note the different ranges for T_1 in (c), (d). Black curves are calculated by Maple 2021 and red curves are fitted by Origin 9 mono- or bi-exponential functions, with lifetimes as indicated.

Singlet and triplet energy transfer.

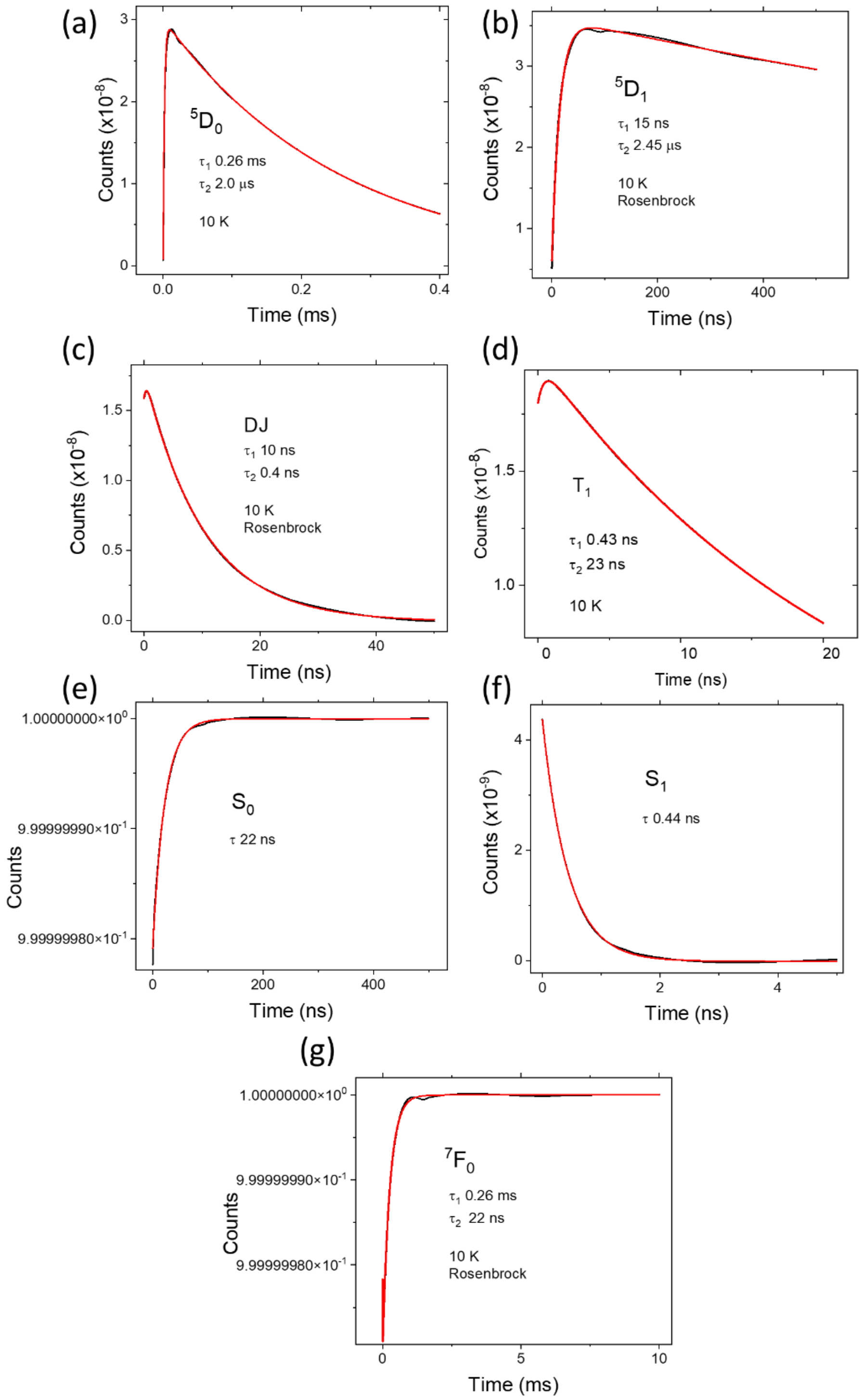

Figure S14. Calculated 10 K time profiles of levels using the Rosenbrock method, following a 5 ns pulse. (a) $^5D_0$; (b) $^5D_1$; (c) DJ; (d) T_1; (e) S_0; (f) S_1; (g) $^7F_0$. The parameters are in columns 4 and 8 of Table 3 (black

lines). Black curves are calculated by Maple 2021 and red curves are fitted by Origin 9 mono- or bi-exponential functions, with lifetimes as indicated.

S8. CALCULATIONS USING THE MODEL OF MALTA

A. Comments upon the model. There are some assumptions in Malta's model - necessary to avoid complicated expressions with parameters unrelated to experimental data and some restrictions on the usage of parameters in the LUMPAC[S26] and JOYSpectra[S27] software. The model employs selection rules based upon *J*-multiplet terms. By contrast, note that the crystal field levels of each multiplet often have mixed parentage and they are split at a low site symmetry over a range of energy. For example, even in a highly symmetric octahedral environment of Eu^{3+} in **$Cs_2NaEuCl_6$**, the $^5L_6$ multiplet is split by 584 cm^{-1}.[S11]

In the theory of Malta, and subsequently in LUMPAC and JOYSpectra, the spectral overlap integral has been represented by *F*, in units of erg^{-1}:

$$F = \frac{1}{\hbar\gamma_L}\sqrt{\frac{ln2}{\pi}}\,e^{-\left(\frac{\Delta}{\hbar\gamma_L}\right)^2 ln2} \qquad \text{(S4)}$$

where γ_L is the full width at half maximum (FWHM) of the broad emission band (rad s^{-1}), $\hbar$ is the reduced Planck constant and Δ (erg) is the energy difference between the band maxima.

For example, as above in Section S6, γ_L = 0.6 eV = 0.6 x 8065.5439 cm^{-1} = 4839.326 cm^{-1};

Δ = 2.77 - 2.14 = 0.63 eV = 5081.293 cm^{-1}.

$$F = \frac{1}{1.054571726\times10^{-34}\times4839.326\times2.99792458\times10^{10}\times2\times\pi}\left[\frac{0.693147}{3.141593}\right]^{0.5} \times exp\left[\left\{-\frac{5081.293}{4839.326}\right\}^2 0.693147\right] = 2.275572\times10^{18}\,J^{-1}$$

F = 2.275572 x 10^{18} J^{-1} ~ 0.365 eV^{-1}.

This value is 4.96 times larger than that calculated from Maple 2021 above. The Table S5 shows calculated values of the overlap integral using direct integration with Maple 2021 and using the formula S4 for *F*. The ratio of the values obtained is less than 5 for values of Δ (ligand energy – Eu^{3+} energy) ~ 0.7 eV ~ 5646 cm^{-1} but increases to a factor near 5000 for values of Δ over 10000 cm^{-1}. In practice, the singlet emission band maximum is usually lower than 3 eV but both LUMPAC[S26] and JOYSpectra[S27] use the energy of the vertical transition (and not the zero phonon line) in the absorption spectrum. In this case, the singlet state peak maximum would be about 0.58 eV (4700 cm^{-1}) too high in the present case for **$Eu(TTA)_3(H_2O)_2$**, leading to considerable error in *F*.

Ligand energy[a]		Spectral overlap integral (eV^{-1})		Ratio A/B
eV	cm^{-1}	(A) Direct calculation	(B)Using formula S4	
2.3	18551	1.2855	0.7452	1.7

2.77	22342	0.0737	0.3646	0.20
3.0	24197	5.26E-3	0.1885	0.028
3.3	26616	4.9E-5	0.0587	8.3E-4
3.4	27423	7.67E-6	0.0368	2.1E-4

Table S5. Calculation of spectral overlap integral using direct integration with Maple 2021 and with Eq. S4 for *F*. The ligand band maximum with FWHM of 0.6 eV is varied in energy whilst keeping the Eu^{3+} level constant at 2.14 eV, with FWHM of 0.006 eV. [a]This is the band maximum, not the zero phonon line energy. Note that the value of γ_L is fixed in calculations of LUMPAC at 3250 cm^{-1} and in JOYSpectra it can be varied.

In fact, in our manuscript, the spectral overlap integrals are all small, in the narrow range from 0.17 eV^{-1} to 2.3 eV^{-1} (column 3, Table 2). The values calculated by Eq. S4 for *F* by using the band maxima for donor emission and acceptor absorption are within an order of magnitude except for singlet to $^5D_0$ ET. Note however that both LUMPAC and JOYSpectra do not use donor emission band maxima but utilize the donor absorption band maxima for calculation and this may be about 8000 cm^{-1} higher in energy than the emission band maximum. The results then differ considerably.

B. Calculations using LUMPAC and JOYSpectra. The software LUMPAC[S26] utilizes the europium room temperature emission spectrum, the $^5D_0$ emission lifetime, and an input file with the molecular structure of the complex. The output gives ET rates from the ligand singlet and triplet states to various Eu^{3+} states. It is described in a tutorial[S28] and in Ref. S20.

Some points concerning the program are that:

(i) the ligand singlet transition dipole strength is fixed at 10^{-35} esu^2 cm^2.

(ii) the donor-acceptor spectral overlap function *F* uses the value of ligand full width at half-maximum (FWHM) equal to 3250 cm^{-1} (0.40 eV).

(iii) LUMPAC considers a thermal population of 0.6 and 0.3 for $^7F_0$ and $^7F_1$, respectively, so that ET for the T_1 - $^5D_0$ channel corresponds to the excitation $^7F_1 \rightarrow$ $^5D_0$.

(iv) The energy of the singlet vertical absorption transition (instead of emission) is employed in the calculation of *F*.

(v) The Eu^{3+} energy levels and matrix elements are taken from the study of LaF_3:Eu^{3+} whereas the coordination sphere of Eu^{3+} in organic complexes usually comprises nitrogen and/or oxygen atoms.

(vi) The calculation of triplet and singlet state energies is fast using INDO/S-CIS or TD-DFT in ORCA but the results are inaccurate.

(vii) The formulation of the rate equations is not the same as in herein.

(viii) The model of Malta includes the contribution from exchange interaction, but the relevant exchange integral is approximated by that of the dipole moment operator. In particular, the same magnitude is taken for singlet or triplet donors.

When calculating the ET rate from the antenna singlet state to Eu^{3+}, both LUMPAC and JOYSpectra[S29] require the energy of the $S_1 \rightarrow S_0$ band maximum. The value in LUMPAC is taken from the energy calculation by ZINDO/S for the singlet state with highest oscillator strength, although the state can be changed by manually modifying the file. The value in JOYSpectra may be entered but for the example file of **Eu(TTA)$_3$(H$_2$O)$_2$** it has been set at 29900 cm^{-1} (334 nm). This energy is far above the S_1 zero phonon line from which transfer occurs.

The more recent JOYSpectra software[S27,S29] overcomes some of these points, in particular in providing the opportunity to adjust the FWHM in the calculation of *F*. The example input file for **Eu(TTA)$_3$(H$_2$O)$_2$**[S29] uses S_1 and T_1 energies 29900 and 20300 cm^{-1}, respectively, which lead to errors since the values are inaccurate. In particular, ET processes are listed for Eu^{3+} levels which are higher in energy than the donor singlet state.

The ET rate calculation was carried out using the coordinates given for **Eu(TTA)$_3$(H$_2$O)$_2$** in JOYSpectra. The ORCA energy level calculation in LUMPAC utilizes ZINDO/S and it gave triplet energies commencing at 12755 cm^{-1}, which is far too low in energy. The normal (auto) calculations in the software choose singlet states which are far above normal excitation wavelengths so that the calculations do not reflect physical situations. For the calculation of ET rates in our case, Table S6, the calculated fourth triplet (19961 cm^{-1}) and lowest singlet state (28964 cm^{-1}) were chosen as the ligand states in LUMPAC. In JOYSpectra, the ligand states were chosen at S_1 25000 cm^{-1}, T_1 19802 cm^{-1}. Our calculations for the ET rates of Eu(TTA)$_3$(H$_2$O)$_2$ using these energies in the software LUMPAC and JOYSpectra are given in Table S6. The overwhelming contribution comes from exchange because of the approximation made for its matrix element.[S30,S31]

**Ligand states**	**Eu^{3+} states**	**Overall ET rate (s^{-1})**				
		LUMPAC<br>γ_L 3250 cm^{-1}	JOYSpectra<br>γ_L 3000 cm^{-1}	JOYSpectra<br>γ_L 4000 cm^{-1}	Ref. S32	Ref. S33
$S_1 \rightarrow S_0$	$^7F_0 \rightarrow {}^5D_0$	3.91E5 ex				
	$^7F_0 \rightarrow {}^5D_1$	2.38E6 ex	7.82E7 ex	1.95E8 ex		
	$^7F_0 \rightarrow {}^5D_4$	1.60E5 mp				3.5E7
	$^7F_0 \rightarrow {}^5G_6$	1.33E4 mp				1.2E8
	$^7F_0 \rightarrow {}^5L_6$	3.18E4 mp			1.37E9	1.4E9
	$^7F_1 \rightarrow {}^5D_0$		2.28E6 ex	1.54E7 ex		

	$^7F_1 \rightarrow {}^5D_1$		1.44E2 dd<br>7.50E5 dm<br>3.76E3 ex	2.20E6 dd<br>4.18E2 dm<br>2.18E6 ex		
	$^7F_1 \rightarrow {}^5D_2$		4.24E6 ex	5.30E6 ex		
	$^7F_1 \rightarrow {}^5D_3$		1.92E3 dd<br>2.48E6 dm	1.49E3 dd<br>1.92E6 dm		
$T_1 \rightarrow T_0$	$^7F_0 \rightarrow {}^5D_0$	9.85E8 ex				4.6E7
	$^7F_0 \rightarrow {}^5D_1$	7.64E8 ex	3.88E8 ex	2.94E8 ex	9.90E8	9.9E8
	$^7F_1 \rightarrow {}^5D_0$		6.11E7 ex	6.06E7 ex	4.60E7	

Table S6. Comparison of our results for ET rates of **$Eu(TTA)_3(H_2O)_2$** using the software LUMPAC and JOYSpectra with those from Refs. 32,33, as described above. (The mechanisms are given as ex – exchange; dd – dipole-dipole; dm – dipole-multipole; mp - multipolar). We consider that ET only occurs from the lowest singlet state, not higher ones. Note that the $T_1 \rightarrow T_0$, $^7F_0 \rightarrow {}^5D_1$ rate is 23 times higher than in Table 3 of the manuscript.

C. Calculations employing the model of Malta but with some parameters taken from the present study.

The aim of the following is to show that simple calculations can be made without recourse to software since many matrix elements are zero.

This Section gives our calculations of ET rates for examples of some transitions in **$Eu(TTA)_3(H_2O)_2$** using the model of Malta, with the spectral overlap integral and dipole strength (calculated from oscillator strength) taken from the present study. In conclusion, the ED-ED transfer rates are calculated in the region of 10^5 s^{-1} but the ED-EQ rate for $S_1 \rightarrow S_0$, $^7F_0 \rightarrow {}^5D_2$ is within an order of magnitude of that calculated in Table 2 of the manuscript for ED-ED transfer.

Reduced spin matrix elements. The squared reduced spin matrix elements of Eu^{3+} have been taken by Malta's group from the free ion wavefunctions of Ofelt.[S34] The ground state $^7F_0$ has four *SLJ* components and other free ion terms have up to ten. Professor Y. Y. Yeung kindly provided to us the free ion compositions, comprising 10 *SLJ* components for each multiplet term, calculated with 6 decimal places from the fits of the energy levels of **$Cs_2NaEuCl_6$**, **LaF_3:Eu^{3+}** and **$NaCdPO_4$:Eu^{3+}** so that we could check if the reduced matrix elements show any dependence upon Eu^{3+} coordination. We employed the formula for the matrix element:

$$< \psi LSJ||S||\psi' L'S'J' > = \sum_i a_i < \psi LSJ||S||\psi' L'S'J' >_i \quad \text{(S5)}$$

Where a_i represents the product of the compositions of initial and final states, and

$$< \psi LSJ||S||\psi' L'S'J' >_i$$

$$= \delta_{\psi,\psi'} \delta_{L,L'} \delta_{S,S'} (-1)^{L+S+J+1} [(2J+1)(2J'+1)S(S+1)(2S+1)]^{1/2} \begin{Bmatrix} S & J & L \\ J' & S & 1 \end{Bmatrix} \quad \text{(S6)}$$

Note the power of (-1). Our results in the Table S7 show generally similar squared matrix elements for the three systems, with oxygen coordination having the lower values, although there are some differences with the tabulation of Ref. 35. The value of the matrix element depends upon the number of intermediate coupled states chosen. For $|<^7F_0||S||^5D_2>|^2$, the 6*j* symbol gives the value zero for our included states, but a value of 0.0009 has been reported.[36]

Table S7. Calculated squared reduced spin matrix elements, $| < \psi LSJ\|S\|\psi' L'S'J' > |^2$.

Squared matrix element	This work			Ref. S35 (From Ref. S34)
	**$Cs_2NaEuCl_6$**	**$LaF_3:Eu^{3+}$**	**$NaCdPO4:Eu^{3+}$**	
$|<^7F_0||S||^5D_1>|^2$	0.02081	0.02097	0.02058	0.0273
$|<^7F_0||S||^5D_2>|^2$	0	0	0	-
$|<^7F_0||S||^5D_3>|^2$	0	0	0	-
$|<^7F_1||S||^5D_0>|^2$	0.11090	0.11182	0.10889	0.117
$|<^7F_1||S||^5D_1>|^2$	1.64882E-7	1.64882E-7	1.15213E-7	2.81E-5
$|<^7F_1||S||^5D_2>|^2$	9.61608E-3	9.62634E-3	9.48934E-3	4.58E-3

Electric dipole-electric dipole energy transfer in $Eu(TTA)_3(H_2O)_2$. The equation given in Ref. S20 for ED-ED ET, with the symbols defined therein, is:

$$W_{d-d} = \frac{S_L(1-\sigma_1)^2}{(2J+1)G} \frac{4\pi}{\hbar} \frac{e^2}{R_L^6} \sum_\lambda \Omega_\lambda^{FED} < \psi' J'||U^{(\lambda)}||\psi J >^2 F \quad \text{(S7)}$$

where: W (s^{-1}) = (S_L (esu^2 cm^2) e^2 (esu^2) F (erg^{-1}) Ω (cm^2))/(h (erg s) R^6 (cm^6))

and we use the values of the dipole strength S_L and F from the present study.

Consider $S_1 \rightarrow S_0$ and $^7F_0 - {}^5L_6$:

e = 4.8032x10^{-10} cm$^{3/2}$g$^{1/2}$s^{-1}; $h/2\pi$ = 1.0546x10^{-27} cm^{2}g s^{-1}; R_L = 4.5x10^{-8} cm; F = 0.172 eV^{-1} = 1.0735x10^{11} erg^{-1}. Take the singlet oscillator strength maximum value 0.05 (*c.f.* Eq. 7):

$$S_L = \frac{3he^2}{8\pi^2 m\nu} g_i \frac{9}{n(n^2+2)^2} P_{if} \sim 1.53\text{x}10^{-35} \text{ esu}^2 \text{ cm}^2 \quad \text{(S8)}$$

The $U^{(\lambda)}$ matrix elements are zero for λ = 2,4 and for λ = 6 it is 0.0153, for $|<^5L_6||U^{(6)}||^7F_0>|^2$.

Ω_λ^{FED} from JOYSpectra are λ = 2, 8.068E-22; λ = 4, 8.246E-22; λ = 6, 1.662E-21 cm^2. We only need λ = 6.

J = 0 for the acceptor level $^7F_0$[S20] and G = 1 for the donor S_1. σ_1 = 0.66917.[S27] So, putting in these numbers, and correcting for the ground state population (say, by the factor of 0.6), W_{ET} ~ 9.08 x 10^5 s^{-1}. The mixing with $^7F_2$ and contribution from $^7F_1$ have not been considered.

Consider $S_1 \rightarrow S_0$ and $^7F_0$ – $^5D_2$:

The $U^{(\lambda)}$ matrix elements are given as zero in JOYSpectra so that the respective ET rates are zero. However, the $U^{(2)}$ matrix element squared has been reported as 0.0009 for oxide systems.[S36] Noting that Ω_2^{FED}= 8.068E-22, and from Table 1, F = 1.335 x 6.241496E11 erg^{-1}, and correcting for the ground state population, W_{ET} ~ 2.01 x 10^5 s^{-1}.

Consider $S_1 \rightarrow S_0$ and $^7F_0$ – $^5D_{0,1}$:

The squared matrix element $| < \psi'J'||U^{(\lambda)}||\psi J > |^2$ is zero for these cases, in the absence of J-state mixing so that the values are zero.

Electric dipole-electric multipole energy transfer in Eu(TTA)$_3$(H$_2$O)$_2$. The equation in Ref. S20, with definitions therein, in cgs units, is:

$$W_{d-m} = \frac{S_L}{(2J+1)G} \frac{2\pi e^2}{\hbar} \sum_\lambda (\lambda+1) \frac{<r^\lambda>^2}{(R_L^{\lambda+2})^2} < f||C^{(\lambda)}||f >^2 (1-\sigma_\lambda)^2 < \psi'J'||U^{(\lambda)}||\psi J >^2 F \quad \text{(S9)}$$

where λ = 2,4,6; the radial integrals: $< r^2 >$ = 0.916 a.u.; $< r^4 >$= 2.017 a.u.; $< r^6 >$= 9.025 a.u.;

$< r^8 >$ = 82.089 a.u, and 1 Bohr = 5.2917721067121 × 10^{-9} cm. The Racah operator integrals: <3||C^2||3 = -1.366; <3||C^4||3> = 1.128; <3||C^6||3> = -1.27. The values of σ_λ are uncertain and are taken from Ref. S32 as σ_2 = 0.6; σ_4 = 0.139; σ_6 = 0.1. The corresponding values in JOYSpectra are 0.512, 0.019 and -0.0308, respectively.

Taking the acceptor level J = 0, the donor state G = 1, in addition to R_L = 4.5 Å, and the value of S_L as above (1.53x10^{-35} esu^2 cm^2), we have, considering singlet ET:

For λ = 2:

$$W_{d-m} = \frac{S_L}{(2J+1)G} \frac{2\pi e^2}{\hbar} (3) \frac{<r^2>^2}{(R_L^4)^2} < 3||C^{(2)}||3 >^2 (1-\sigma_2)^2 < \psi'J'||U^{(2)}||\psi J >^2 F \quad \text{(S10a)}$$

$$W_{d-m} = 0.737026 < \psi'J'||U^{(2)}||\psi J >^2 F \quad \text{(S10b)}$$

For λ= 4:

$$W_{d-m} = \frac{S_L}{(2J+1)G} \frac{2\pi e^2}{\hbar} (5) \frac{<r^4>^2}{(R_L^6)^2} < 3||C^{(4)}||3 >^2 (1-\sigma_4)^2 < \psi'J'||U^{(4)}||\psi J >^2 F \quad \text{(S11a)}$$

$$W_{d-m} = 3.598 \times 10^{-3} < \psi'J'||U^{(4)}||\psi J >^2 F \quad \text{(S11b)}$$

For λ= 6:

$$W_{d-m} = \frac{S_L}{(2J+1)G} \frac{2\pi e^2}{\hbar} (7) \frac{<r^6>^2}{(R_L^8)^2} < 3||C^{(6)}||3 >^2 (1-\sigma_6)^2 < \psi'J'||U^{(6)}||\psi J >^2 F \quad \text{(S12a)}$$

$$W_{d-m} = 2.671 \times 10^{-5} < \psi'J'||U^{(6)}||\psi J >^2 F \quad \text{(S12b)}$$

so that the ratios of $W_{d\text{-}m}$ are 1:4.9x10^{-3}:3.6x10^{-5} for λ = 2,4,6, assuming equal squared reduced matrix elements and overlap integrals in Eqs. S10b, S11b and S12b. Alternatively, taking the values above of σ_i (i = 2,4,6) as in JOYSpectra, the numerical values in Eqs. S10b-S12b are 1.09699, 4.67134x10^{-3} and 3.50435x10^{-5}, respectively, with the ratio of 1:4.3x10^{-3}:3.2x10^{-5}, so that there is not a great deal of difference. In the following, we employ the values of σ_i from Ref. S32.

Consider $S_1 \rightarrow S_0$ and $^7F_0$ – $^5L_6$:

In Table S6, the largest values of $W_{d\text{-}m}$ for the $^7F_0$ acceptor state are for $^5L_6$, $^5D_4$ and $^5G_6$. We do not consider the latter two because their energies lie above $S_1 \rightarrow S_0$. For $^5L_6$, the squared $U^{(\lambda)}$ matrix elements are zero for λ = 2,4 and for λ = 6 the value is 0.0153. The value of the spectral overlap integral in Table 1 is 1.0735x10^{11} erg^{-1}, so that the calculated multipolar ET rate after correcting for the population of $^7F_0$, is 2.63x10^{4} s^{-1}, without back-transfer.

Consider $S_1 \rightarrow S_0$ and $^7F_0$ – $^5D_2$:

For $^7F_0 \rightarrow {}^5D_2$, $[U^{(2)}]^2$ = 0.0009, F = 8.332397 x 10^{11} erg^{-1}, so that from S10(b), correcting for $^7F_0$ population, W_{ET} ~ 3.32 x 10^{8} s^{-1}.

Energy transfer in $Eu(TTA)_3(H_2O)_2$ by the exchange mechanism. The formula given in Ref. S20, with definitions, for the exchange mechanism is:

$$W_{ex} = \frac{(1-\sigma_0)^2}{(2J+1)G}\frac{8\pi}{3\hbar}\frac{e^2}{R_L^4} < \psi'J'||S||\psi J >^2 \, e^2 \sum_m | < \Psi_{N-1}\Pi| \sum_j r_j C_0^{(1)}(j) s_{-m}(j) |\Psi_{N-1}\Pi^* > |^2 F \qquad \text{(S13a)}$$

and it has been approximated as:[S20]

$$W_{ex} = \frac{(1-\sigma_0)^2}{(2J+1)G}\frac{8\pi}{3\hbar}\frac{e^2}{R_L^4} < \psi'J'||S||\psi J >^2 \frac{S_L'}{4} F \qquad \text{(S13b)}$$

Where S_L' is the dipole strength of the ligand transition. The value of σ_0 in JOYSpectra is 0.99288. Taking R_L = 4.5 Å, J = 0 and G = 3 for triplet ET, we have:

$$W_{ex} = 1.8881 \times 10^{33} < \psi'J'||S||\psi J >^2 S_L'F \qquad \text{(S14)}$$

In Malta's original paper,[S37] in a slightly modified equation, the squared matrix element was taken as 0.04, the dipole strength 10^{-36} esu^2 cm^2, R_L 4 Å, σ_0 = 0. Furthermore, the values γ_L = 5000 cm^{-1} and Δ = 1000 cm^{-1} lead to F = 4.6x10^{11} erg^{-1}. Using his Eq. 11 and 12 in Ref. S37, we calculate W_{ET} = 1.3x10^{13} s^{-1}, and 7.8x10^{12} s^{-1} allowing for the population of $^7F_0$, compared to his statement that the ET is of the order 10^{12} s^{-1}. This value is too high.

Consider $T_1 \rightarrow S_0$ and $^7F_0 \rightarrow {}^5D_1$ exchange ET

Taking the triplet oscillator strength as in Table 2 (S_L ~ 2.0x10^{-42} esu^2 cm^2), F = 2.29 eV^{-1} ~ 1.429x10^{12} erg^{-1}, $|<^5D_1||S||^7F_0>|^2$ = 2.73x10^{-2},[S35] we obtain W_{ET} = 147 s^{-1}. It is strange to obtain such a small answer, compared to the above and to Table S6 (~10^{8} s^{-1}). Clearly, the value of S_L' in this case is much larger than just taking the value S_L from the triplet oscillator strength.

In JOYSpectra,[S29] the value of S_L' has been taken as 10^{-36} esu^2 cm^2. This would give the ET rate 7.4x10^7 s^{-1} in Eq. S14, using F = 1.429x10^{12} erg^{-1}, $|<^5D_1||S||^7F_0>|^2$ = 2.73x10^{-2}. This ET rate is similar to the experimental value derived in the main text for the ET transition $T_1 \rightarrow S_0$ and $^7F_0 \rightarrow {}^5D_1$ (2.84x10^7 s^{-1}).

In conclusion, some of the approximations made in calculations employing the model of Malta have been highlighted, particularly with respect to the overlap integral F, the magnitude of the exchange integral, and the use of incorrect transition energies. It has also been demonstrated that such calculations can easily be performed by hand. For a discussion of the use of forced electric dipole (static coupling) and dynamic coupling matrix elements, and their cross-terms, for Eu^{3+} complexes, the reader is referred to Refs. S14 and S16.

S9. UNITS AND CONVERSIONS USED IN THIS WORK

a_0 = 5.291772…x10^{-11} m. Bohr radius

α = 7.297 352 5693 x 10^{-3}. Fine structure constant

k_B = 0.6950356 cm^{-1} K^{-1}. Boltzmann constant

h = 6.62607004× 10^{-34} J s. Planck constant (kg m^2 s^{-1})

$\hbar$ = 1.0545718x10^{-34} J s, 6.58211956x10^{-16} eV s

m = 9.10938356×10^{-31} kg, electron mass

e = 1.6021766208×10^{-19} C, electron charge (A s)

c = 2.99792458×10^8 m s^{-1}, the speed of light

ε_0 = 8.854187817×10^{-12} F m^{-1}, the vacuum permittivity (kg^{-1} m^{-3} s^4 A^2)

P_x oscillator strength of X , dimensionless

1 eV = 1239.84193 nm; 1 eV = 1.60218x10^{-19} J; 1 eV = 8065.54429 cm^{-1}

1 eV^{-1} = 6.2415x10^{11} erg^{-1}; 1 eV = 1.60218x10^{-12} erg

1 cm^{-1} = 1.98644587x10^{-23} J = 1.239841984x10^{-4} eV

1 s^{-1} = 4.13558x10^{-15} eV

1 J = 10^7 erg, 1 J^{-1} = 10^{-7} erg^{-1}

L = 6.02214076x10^{23} mol^{-1}

ESU unit statcoulomb, 1 statC [cm$^{3/2}$ g$^{1/2}$ s^{-1}]. For electric charge, 1 C = 2.997924580x10^9 statC, and 1 statC = 3.335641x10^{-10} C and 1 cm = 10^{-2} m, so that 1 esu^2 cm^2 = (3.335641x10^{-10} C)^2 x (10^{-2} m)^2 = 1.112650x10^{-23} C^2 m^2, or 1 C^2 m^2 = 8.987552x10^{22} esu^2 cm^2.

S10. REFERENCES

(S1) Charles, R. G.; Ohlmann, R. C. Europium Thenoyltrifluoroacetate, Preparation and Fluorescence Properties. *J. Inorg. Nucl. Chem.* **1965**, *27*, 255-259.

(S2) Fernandes, M.; de Zea Bermudez, V.; Sá Ferreira, R. A.; Carlos, L. D.; Charas, A.; Morgado, J.; Silva, M. M.; Smith, M. J. Highly Photostable Luminescent Poly(ε-caprolactone)siloxane Biohybrids Doped with Europium Complexes. *Chem. Mater.* **2007**, *19,* 3892-3901.

(S3) White, J. G. The Crystal Structure of Europium Tris[4,4,4,-trifluoro-1-(2-thienyl)-1,3-butanedione] Dihydrate, *Inorg. Chim. Acta* **1976**, *16,* 159-162.

(S4) Vallet, V.; Fischer, A.; Szabó, Z.; Grenthe, I. The Structure and Bonding of Y, Eu, U, Am and Cm Complexes as Studied by Quantum Chemical Methods and X-Ray Crystallography. *Dalton Trans*. **2010**, *39*, 7666-7672.

(S5) Zhang, Y.; Thor, W.; Wong, K.-L.; Tanner, P. A. Determination of Triplet State Energy and the Absorption Spectrum for a Lanthanide Complex. *J. Phys. Chem. C* **2021**, *125*, 7022-7033.

(S6) Zhou, L.; Zhang, H.; Deng, R.; Li, Z.; Yu, J.; Guo, Z. Conversion Process of the Dominant Electroluminescence Mechanism in a Molecularly Doped Organic Light-Emitting Device with Only Electron Trapping. *J. Appl. Phys.* **2007**, *102*, 064504.

(S7) Zucchi, G.; Murugesan, V.; Tondelier, D.; Aldakov, D.; Jeon, T.; Yang, F.; Thuéry, P.; Ephritikhine, M.; Geffroy, B. Solution, Solid State, and Film Properties of a Structurally Characterized Highly Luminescent Molecular Europium Plastic Material Excitable with Visible Light. *Inorg. Chem*. **2011**, *50*, 4851-4856.

(S8) Thor, W.; Wu, Y.; Wang, L.; Zhang, Y.; Tanner, P. A.; Wong, K.-L. Charging and Ultralong Phosphorescence of Lanthanide Facilitated Organic Complex. *Nat. Commun*. **2021**, *12*, 6532.

(S9) Chang, N. C.; Gruber, J. B. Spectra and Energy Levels of Eu^{3+} in Y_2O_3, *J. Chem. Phys.* **1964**, *41*, 3227-3234.

(S10) Binnemans, K.; Görller-Walrand, C. Crystal Field Analysis of $EuCl_3.6H_2O$. *J. Alloys Compd.* **1997**, *250,* 326-331.

(S11) Thorne, J. R. G.; Jones, M.; McCaw, C. S.; Murdoch, K. M.; Denning, R. G.; Khaidukov, N. M. Two-Photon Spectroscopy of Europium(III) Elpasolites. *J. Phys. Cond. Matter* **1999**, *11*, 7851-7866.

(S12) Carnall, W. T.; Crosswhite, H.; Crosswhite, H. M. Energy Level Structure and Transition Probabilities in the Spectra of the Trivalent Lanthanides in LaF_3. Report ANL-78-XX-95. doi:10.2172/6417825.

(S13) Hellwege, K. H.; Kahle, H. G. Specktrum und Struktur Kristalliner Europiumsalze. *Z. Physik* **1951**, *129*, 62-84.

(S14) Kirby, A. F.; Richardson, F. S. Detailed Analysis of the Optical Absorption and Emission Spectra of Eu^{3+} in the Trigonal (C_3) $Eu(DBM)_3.H_2O$ System. *J. Phys. Chem.* **1983**, *87*, 2544-2556.

(S15) Kirby, A. F.; Foster, D.; Richardson, F. S. Comparison of $^7F_J \leftarrow ^5D_0$ Emission Spectra for Eu(III) in Crystalline Environments of Octahedral, Near-Octahedral, and Trigonal Symmetry. *Chem. Phys. Lett.* **1983**, *95*, 507-512.

(S16) Kirby, A. F.; Richardson, F. S. Optical Excitation and Emission Spectra of Eu^{3+} in Microcrystalline Samples of Trigonal $Na_3[Eu(ODA)_3].2NaClO_4.6H_2O$. *J. Phys. Chem.* **1983**, *87*, 2557-2563.

(S17) Ferrier, A.; Tumino, B.; Ph. Goldner, Variations in the Oscillator Strength of the $^7F_0 \rightarrow {}^5D_0$ Transition in $Eu^{3+}:Y_2SiO_5$ Single Crystals. *J. Lumin.* **2016**, *170*, 406-410.

(S18) Maple 2021. Maplesoft, A Division of Waterloo Maple Inc., Waterloo, Ontario.

(S19) Origin(Pro), Version Number 9. OriginLab Corporation, Northampton, MA, USA.

(S20) Carneiro Neto, A. N.; Teotonio, E. E. S.; de Sá, G. F.; Brito, H. F.; Legendziewicz, J.; Carlos, L. D.; Felinto, M. C. F. C.; Gawryszewska, P.; Moura Jr., R. T.; Longo, R. L.; et al. Modeling Intramolecular Energy Transfer in Lanthanide Chelates: A Critical Review and Recent Advances. *Handbook on the Physics and Chemistry of Rare Earths*, **2019**, *56*, 55-162.

(S21) Faustino, W. M.; Nunes, L. A.; Terra, I. A. A.; Felinto, M. C. F. C.; Brito, H. F.; Malta, O. L. Measurement and Model Calculation of the Temperature Dependence of Ligand-to-Metal Energy Transfer Rates in Lanthanide Complexes. *J. Lumin*. **2013**, *137*, 269-273.

(S22) Malkin, B. Z.; Pukhov, K. K.; Saikin, S. K.; Baibekov, E. I.; Zakirov, A. R. Theoretical Studies of Nonradiative 4f–4f Multiphonon Transitions in Dielectric Crystals Containing Rare Earth Ions. *Journal of Molecular Structure.* **2007**, *838*, 170-175.

(S23) De Mello Donegá, Meijerink, A.; Blasse, G. Non-Radiative Relaxation Processes of the Pr^{3+} Ion in Solids. *J. Phys. Chem. Solids.* **1995**, *56*, 673-685.

(S24) German, K. R.; Kiel, A. Radiative and Nonradiative Transitions In $LaCl_3:Pr^{3+}$ and $PrCl_3$, *Phys. Rev. B* **1973**, *8*, 1846-1853.

(S25) Reed Jr., E. D.; Moos, H. W. Nonthermalization and Large Variation in Multiphonon Relaxation Rate among Rare-Earth-Ion Stark Levels. *Phys. Rev. B* **1973**, *8*, 988-992.

(S26) Dutra, J. D. L.; Bispo, T. D.; Freire, R. O. LUMPAC Lanthanide Luminescence Software Package. *J. Comput. Chem.* **2014**, *35*, 772-775.

(S27) Moura Jr, R. T.; Carneiro Neto, A. N.; Aguiar, E. C.; Santos-Jr., C. V.; de Lima, E. M.; Faustino, W. M.; Teotonio, E. E. S.; Brito, H. F.; Felinto, M. C. F. C.; Ferreira, R. A. S.; et al. JOYSpectra: A Web Platform for Luminescence of Lanthanides. *Opt. Mater. X* **2021**, *11*, 100080.

(S28) LUMPAC Lanthanide Luminescence Software Package. https://lumpac.pro.br/ (Accessed on 18 August 2022).

(S29) JOYSpectra a Web Platform for Luminescence of Lanthanides. www.cca.ufpb.br/joymaker. (Accessed on 18 August 2022).

(S30) Malta, O. L. Ligand-Rare-Earth Ion Energy Transfer in Coordination Compounds. A Theoretical Approach. *J. Lumin*. **1997**, *71*, 229-236.

(S31) Dexter, D. L. A Theory of Sensitized Luminescence in Solids. *J. Chem. Phys.* **1953***, 21*, 836-850.

(S32) Malta, O. L.; Gonçalves e Silva, F. R. A Theoretical Approach to Intramolecular Energy Transfer and Emission Quantum Yields in Coordination Compounds of Rare Earth Ions. *Spectrochim. Acta Part A Mol. Biomol. Spectrosc.* **1998**, *54*, 1593-1599.

(S33) Malta, O. L.; Brito, H. F.; Menezes, J. F. S.; Gongalves e Silva, F. R.; Alves Jr., S.; Farias Jr., F. S.; de Andrade, A. V. M. Spectroscopic Properties of a New Light-Converting Device Eu(Thenoyltrifluoroacetonate), 2(Dibenzyl Sulfoxide). A Theoretical Analysis Based on Structural Data Obtained from a Sparkle Model. *J. Lumin.* **1997**, *75*, 255-268.

(S34) G. S. Ofelt, f^6 Configuration of Rare-earth Ions. *J. Chem. Phys.* **1963**, 38, 2171-2180.

(S35) Kasprzycka, E.; Carneiro Neto, A. N.; Trush, V. A.; Jerzykiewicz, L.; Amirkhanov, V. M.; Malta, O. L.; Legendziewicz, J.; Gawryszewska, P. How Minor Structural Changes Generate Major Consequences in Photophysical Properties of RE Coordination Compounds; Resonance Effect, LMCT State. *J. Rare Earths*. **2020**, *38*, 552-563.

(S35) Kasprzycka, E.; Carneiro Neto, A. N.; Trush, V. A.; Jerzykiewicz, L.; Amirkhanov, V. M.; Malta, O. L.; Legendziewicz, J.; Gawryszewska, P. How Minor Structural Changes Generate Major Consequences in Photophysical Properties of RE Coordination Compounds; Resonance Effect, LMCT State. *J. Rare Earths*. **2020**, *38*, 552-563.

(S36) Ćirića, A.; Stojadinovića, S.; Brik, M. G.; Dramićanin, M. D. Judd-Ofelt Parametrization from Emission Spectra: The Case Study of the Eu^{3+} $^5D_1$ Emitting Level. *Chem. Phys.* **2020**, *528*, 110513 (1–7).

(S37) e Silva, F. R. G.; Malta, O. L. Calculation of the Ligand–Lanthanide Ion Energy Transfer Rate in Coordination Compounds: Contributions of Exchange Interactions. *J. Alloys. Compd.* **1997**, *250*, 427–430.
